# Activation of the TOR Signalling Pathway by Glutamine Regulates Insect Fecundity

**DOI:** 10.1038/srep10694

**Published:** 2015-05-29

**Authors:** Yifan Zhai, Zhongxiang Sun, Jianqing Zhang, Kui Kang, Jie Chen, Wenqing Zhang

**Affiliations:** 1 State Key Laboratory of Biocontrol and School of Life Sciences, Sun Yat-sen University, Guangzhou 510275, China; 2 Institute of Plant Protection, Shandong Academy of Agricultural Sciences, Jinan 250100, China

## Abstract

The target of rapamycin (TOR) positively controls cell growth in response to nutrients such as amino acids. However, research on the specific nutrients sensed by TOR is limited. Glutamine (Gln), a particularly important amino acid involved in metabolism in organisms, is synthesised and catalysed exclusively by glutamine synthetase (GS), and our previous studies have shown that Gln may regulate fecundity in *vivo* levels of the brown planthopper (BPH) *Nilaparvata lugens.* Until now, it has remained unclear whether Gln activates or inhibits the TOR signalling pathway. Here, we performed the combined analyses of iTRAQ (isobaric tags for relative and absolute quantification) and DGE (tag-based digital gene expression) data in *N.lugens* at the protein and transcript levels after GS RNAi, and we found that 52 pathways overlap, including the TOR pathway. We further experimentally demonstrate that Gln activates the TOR pathway by promoting the serine/threonine protein kinase AKT and inhibiting the 5′AMP-activated protein kinase AMPK phosphorylation activity in the pest. Furthermore, TOR regulates the fecundity of *N. lugens* probably by mediating vitellogenin (Vg) expression. This work is the first report that Gln activates the TOR pathway in *vivo*.

In the early 1990s, the target of rapamycin (TOR) was initially discovered in *Saccharomyces cerevisiae*^1^. Since then, TOR has been identified in many other organisms ranging from yeast to mammals^2^,^3^. TOR is an atypical serine/threonine kinase of the phosphatidylinositol kinase-related kinase (PIKK) family and is a major regulator of growth in eukaryotes. TOR is found in two conserved complexes termed the TOR complex 1 (TORC1) and the TOR complex 2 (TORC2)^4^. The TOR pathway is activated by many extra- and intracellular signals such as nutrients (amino acids) and growth factors (insulin). Conversely, TOR is inhibited by numerous stress conditions such as cellular energy (ATP) depletion and hypoxia stress^5^,^6^,^7^.

TOR positively controls cell growth in response to nutrients. However, research on the specific nutrients sensed by TOR is limited^8^. Arginine (Arg) and Leucine (Leu) have an essential role in the phosphorylation of ribosomal S6 kinase (S6 K) and the eukaryotic initiation factor 4E binding protein 1 (4EBP1) mediated by TORC1^9^,^10^. Glutamine (Gln) is the most abundant nonessential amino acid, and its synthesis is catalysed exclusively by glutamine synthetase (GS). Gln regulates the proliferation of diverse cell types, which is a precursor for α-ketoglutarate of the tricarboxylic acid (TCA) cycle, and provides nitrogen for protein and nucleotide synthesis^11^. However, the role of Gln in TOR activation remains poorly defined^12^. In *S. cerevisiae*, Gln controls TOR readouts (8). Similarly in mammalian cells, Gln activates TORC1 signalling^13^, Leu and Gln activate glutaminolysis and mTORC1^12^, and L-glutamine flux regulates mTOR, translation and autophagy to coordinate cell growth and proliferation^14^. In contrast, the transcription factor FOXO3-induced GS activity inhibits mTOR activation by blocking the translocation of mTOR to the lysosomal surface^15^. Thus, it remains unclear whether Gln activates or inhibits the TOR signalling pathway.

Here, we demonstrate that Gln activates the TOR pathway in the brown planthopper (BPH) *Nilaparvata lugens* (Stål), a serious pest of rice crops with robust fecundity^16^. We also show that Gln promotes the serine/threonine protein kinase AKT and inhibits the 5′AMP-activated protein kinase AMPK phosphorylation activity. Furthermore, TOR regulates the fecundity of *N. lugens* probably by mediating vitellogenin (Vg) expression. This study is the first report to show that Gln activates the TOR pathway *in vivo*.

## Results

### Global changes at the protein and transcript levels after GS RNAi

**Figure 1:**
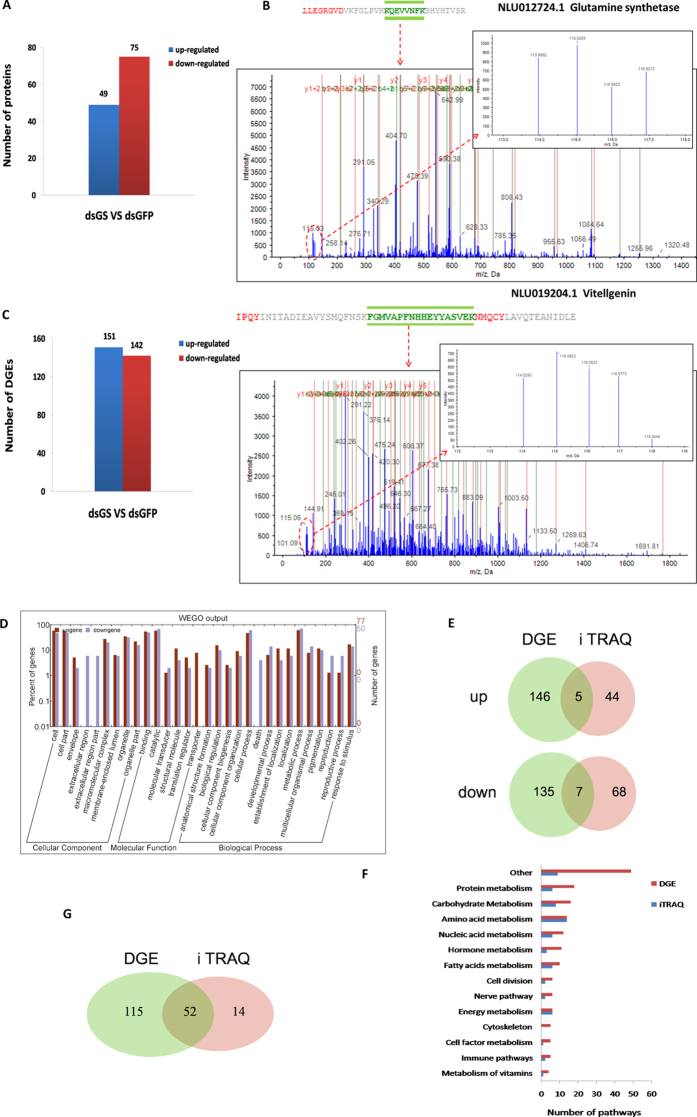
Combined analyses of the iTRAQ and DGE data after GS RNAi. First day brachypterous female adults were injected with ds*GS* or ds*GFP*. Samples were used for DGE and iTRAQ 48 h and 72 h post-injection, respectively. (**A**) No. of differentially expressed proteins identified by iTRAQ, the conditions for protein spots were ≥1.2-fold or ≤0.8 (p < 0.05) for up-regulated or down-regulated proteins in ds*GS* compared to ds*GFP*. (**B**) The representative MS/MS spectra of down-regulated proteins, GS (NLU012724.1) and Vg (NLU019204.1). (**C**) No. of differentially expressed unigenes identified by DGE, the conditions for unigenes was FDR ≤0.001 and |log_2_Ratio| ≥ 1. (**D**) GO analysis of differentially expressed genes. (**E**) Venn diagrams of differentially expressed genes/proteins from iTRAQ and DGE analyses. (**F**) Cluster of pathways for iTRAQ and DGE. (**G**) Venn diagrams of regulated pathways from iTRAQ and DGE analyses.

To ensure that GS knockdown was effective in the 2 samples, the mRNA expression of the GS gene was detected by qRT-PCR (Supplementary Figure 1). Afterwardsthe iTRAQ Mass-Tagging mass spectrometric results from the two repetitions of the GS-RNAi and GFP-RNAi experiments were analysed using the ProteinPilot^TM^ software 2.0.1 (Applied Biosystems, USA). *N. lugens* lacks a reference genome; therefore, the annotation of its proteins primarily depends on its EST databases and other insect databases^17^. This analysis produced a quantitative proteome of 958 proteins for the GS knockdown experiment (Table 1). The GS knockdown experiment resulted in 124 proteins that were statistically significant responders (*p* < 0.01), which is approximately 12.94% of the identified quantitative proteome. Among these proteins, 49 and 75 proteins were up- and down-regulated, respectively (Fig. 1A, Supplementary Table 1). Regarding the down-regulated proteins, we found the RNAi target protein GS (NLU012724.1) and fecundity-related protein vitellogenin (Vg) (NLU019204.1) (Fig. 1B), indicating that the RNAi was effective. Several TOR pathway proteins of interest, including two key upstream activators of Rheb (NLU021861.1) and 3′-phosphoinositide-dependent kinase (PDK1) (NLU017432.3), were also down-regulated.

The differentially expressed genes following RNAi treatment were analysed using the Digital Gene Expression (DGE) method. The results revealed 293 genes with significantly differential expression between the GS-RNAi and GFP-RNAi libraries, including 151 up- and 142 down-regulated genes (Fig. 1C). In the down-regulated genes, we also found GS and Vg in addition to several TOR pathway genes such as TOR (NLU007095.1), Rheb and S6 K (NLU007225.2). According to the GO classification, most of the differentially expressed genes correlated to biological processes, including the reproductive process and various developmental processes (Fig. 1D).

With the iTRAQ and DGE high-quality data sets, we performed an integrated analysis of the proteome and transcriptome. The overall analysis of the robustly regulated genes at the protein and mRNA levels found 5 genes that overlap in the up-regulated group and 7 genes in the down-regulated group (Fig. 1E, Supplementary Table 2). Pathway enrichment analysis for the proteins and transcripts was also performed using the KEGG database. In addition to the “unable to classify” group, the protein metabolism, carbohydrate metabolism and amino acid metabolism groups were ranked in the top 3 most enriched pathway ontology groups (Fig. 1F). Fifty-two overlapping pathways were found between iTRAQ and DGE (Fig. 1G), including the TOR signalling pathway.

### Glutamine activates the TOR pathway *in vivo*

**Figure 2:**
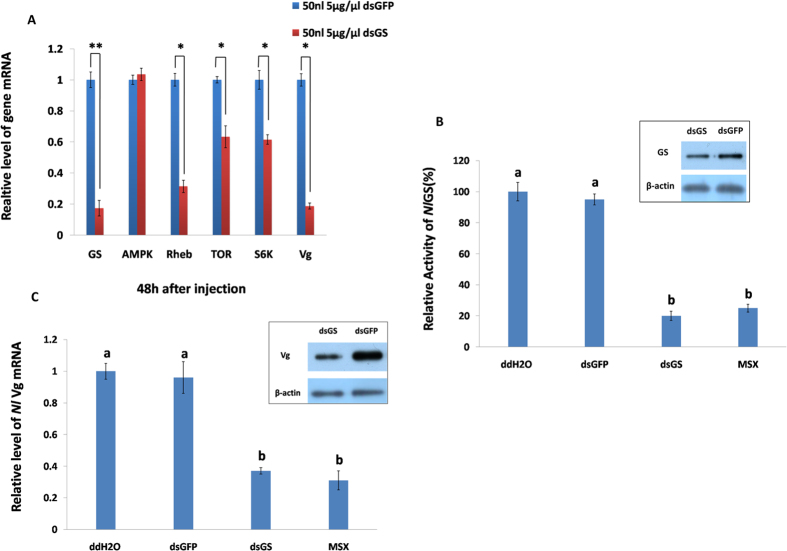
Effect of GS knockdown on gene expression and enzyme activity. First day brachypterous female adults were injected with 50 nL ds*GS* (5 ng/nL), ds*GFP* (5 ng/nL), ddH_2_O or MSX (10 mM), respectively. (**A**) The transcript levels of genes after injection by qRT-PCR. Data represent mean values±S.E.M (n = 3), *p < 0.05, **p < 0.01. (**B**) The relative activity of GS at 48 h post-injection. Data represent mean values±S.E.M (n = 3), and those in the columns followed by the different letters mean significant difference (*p* = 0.05, Duncan’s multiple range test). Inset shows Western blotting analysis of GS protein levels injected with either *dsGFP* or *dsGS*; the *β-*actin gene was used as an internal control. (**C**) The transcript levels of Vg at 48 h post-injection by qRT-PCR. Data represent mean values±S.E.M (n = 3), and those in the columns followed by the different letters mean significant difference (*p* = 0.05, Duncan’s multiple range test). Inset shows Western blotting analysis of Vg protein levels injected with either ds*GFP* or ds*GS*; the *β*-actin gene was used as an internal control.

To validate the above result suggesting that GS knockdown influences the TOR signalling pathway, several experiments were conducted. After studying the feature of GS (Supplementary Figure 2), we depleted GS for 48 h and detected the transcript change of the TOR pathway-associated genes. The RNAi was effective because the GS mRNA and protein levels as well as enzymatic activity decreased (Fig. 2A,B). Three TOR pathway-associated genes, Rheb, TOR and S6 K, had significantly lower expression levels after GS RNAi compared to the control (ds*GFP*) (Fig. 2A), indicating that a positive relationship exists between GS and TOR.

**Figure 3:**
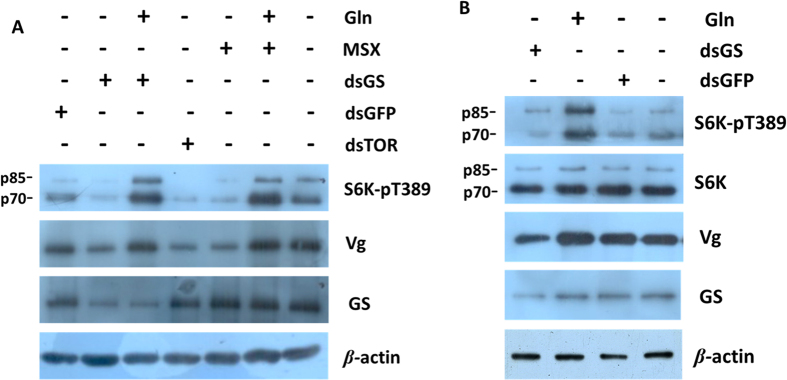
Western blots showing S6 K phosphorylation levels and GS and Vg protein abundance under various nutritional conditions at 48 h post-injection. (**A**) First day brachypterous female adults were injected with 50 nL ds*GFP* (5 ng/nL), ds*GS*, ds*TOR*, MSX (10 mM), Gln (20 mM) and ddH_2_O. Shown are representative blots (n = 3). (**B**) The 3^rd^ to the 5^th^ instar nymphs were fed an artificial diet (w/w Gln) together with ds*GS* or ds*GFP* (0.5 μg/μl). Shown are representative blots (n = 3).

Secondly, we detected the phosphorylation of S6 K, a molecular marker of TOR pathway activation^18^. After injecting 250 ng ds*TOR* or 10 mM rapamycin into the BPH adults, the phosphorylation of S6 K decreased (Supplementary Figure 3). Similar results were observed with the injection of ds*GS* or L-methionine sulfoximine (MSX), a GS specific inhibitor^8^ (Fig. 3A, lane 2 and 5). When Gln (the GS specific product) was added into ds*GS* or MSX, S6 K phosphorylation increased (Fig. 3A, lane 3 and 6). When feeding the BPH nymphs with the artificial diet (with Gln)^19^, S6 K phosphorylation increased compared to the diet without Gln (Fig. 3B, lane 2 and 4). When ds*GS* was added into the diet, S6 K phosphorylation also decreased (Fig. 3B, lane 1). These results show that Gln activates the TOR pathway via the phosphorylation of S6 K *in vivo*. In addition, the Vg mRNA and protein levels were down-regulated after the injection of ds*GS* and MSX (Fig. 2C), indicating that GS regulates BPH fecundity^20^.

### TOR regulates fecundity in BPH

**Figure 4:**
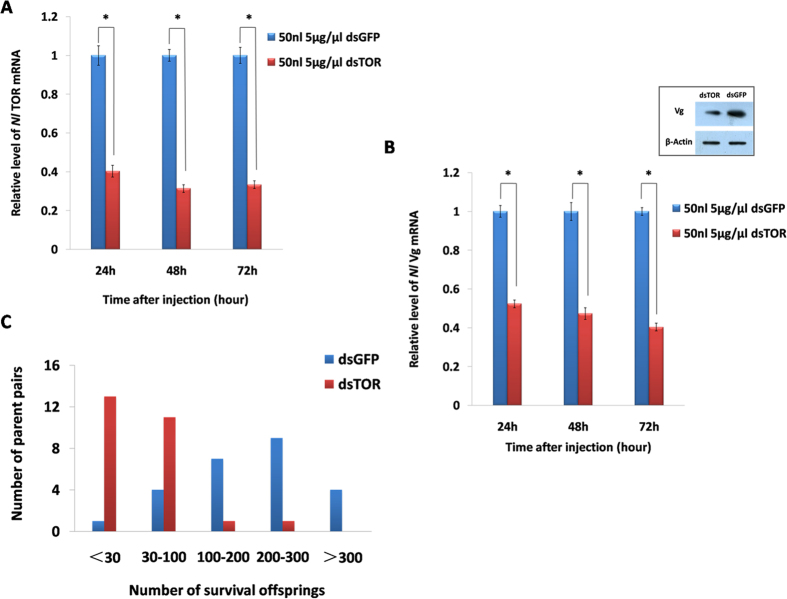
The effect of TOR on fecundity in *N. lugens.* First day brachypterous female adults were injected with ds*TOR* or ds*GFP.* (**A**) The transcript levels of TOR after injection with dsRNA. (**B**) The transcript levels of Vg after RNAi. Data represent the mean values ± S.E.M of three replicates, ‘*’ indicates statistically significant difference (t-test, *p* < 0.05). The inset shows Western blotting analysis of Vg protein levels, and the *β*-actin gene was used as an internal control. (**C**) The frequency distribution of *N. lugens* offspring.

After the injection of ds*TOR* into the BPH adults, the *TOR* mRNA level significantly decreased by 59.66%, 68.66% and 66.68% at 24 h, 48 h and 72 h, respectively, compared to the animals injected with ds*GFP* (Fig. 4A). The expression level of the Vg gene was also significantly down-regulated by 47.66%, 52.74% and 59.68% after ds*TOR* injection. When the BPH adults were sampled 48 h after injectedion of rapamycin or *dsTOR*, significant reductions in the Vg protein level was observed (Fig. 4B, Supplementary Figure 3). As a result, emerged brachypterous BPH adults had fewer offspring (53.22 per pair, a 71.21% decrease). Most ds*TOR*-treated adults had less than 100 offspring; however, 200-300 nymphs were observed for the majority of the ds*GFP*-treated pairs (Fig. 4C). These results suggest that TOR regulates the fecundity of *N. lugens* probably by mediating Vg expression.

### Gln promotes AKT and inhibits AMPK

**Figure 5:**
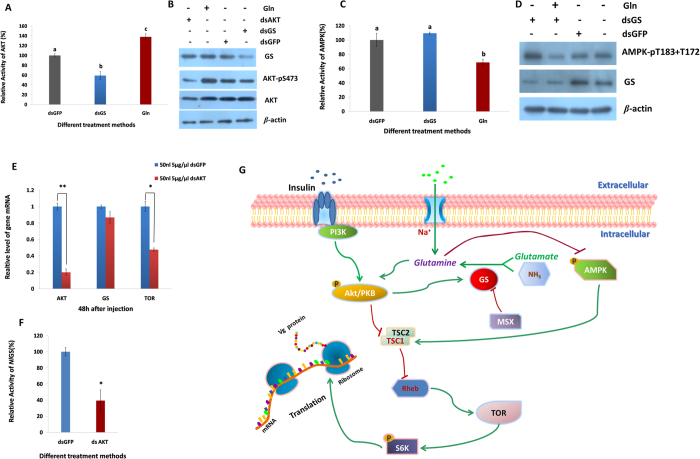
Proposed mechanism of glutamine-mediated activation of the TOR signalling pathway . (**A**) The relative activity of AKT at 48 h post-injection. Data represent the mean values±S.E.M (n = 3), and the values in the columns followed by different letters note a significant difference (*p* = 0.05, Duncan’s multiple range test). (**B**) Western blots showing S6 K phosphorylation levels and GS protein abundance. Shown are representative blots (n = 3). (**C**) The relative activity of AMPK at 48 h post-injection. Data represent the mean values±S.E.M (n = 3), and the values in the columns followed by the different letters note a significant difference (*p* = 0.05, Duncan’s multiple range test). (**D**) Western blots showing S6K phosphorylation levels and GS protein abundance. Shown are representative blots (n = 4). (**E**) The mRNA expression levels at 48 h after injection of ds*AKT*. Data represent the mean values±S.E.M (n = 3), *p < 0.05, **p < 0.01. (**F**) The relative activity of GS at 48 h after injection of ds*AKT*. Data represent the mean values±S.E.M (n = 3), *p < 0.05. (**G**) Proposed model of the activation of the TOR signalling pathway by Gln by promoting AKT and inhibiting AMPK in *N. lugens*.

To further study the molecular mechanism of the activation of the TOR pathway via Gln, we chose two key upstream genes of the TOR pathway, AKT and AMPK. After silencing GS through RNAi, both the enzyme activity and phosphorylation of AKT decreased. When Gln was added, the enzyme activity and phosphorylation of AKT were up-regulated (Fig. 5A,B), suggesting that Gln promoted AKT. However, the opposite trend was observed when AMPK was measured (Fig. 5C,D), indicating that Gln inhibited AMPK.

When AKT was knocked down using RNAi, the TOR mRNA level significantly decreased (Fig. 5E). More importantly, GS activity decreased by 60.65% (Fig. 5F); however, its mRNA level did not significantly change (Fig. 5E), implying that AKT has a positive feedback mechanism that acts on GS.

In conclusion, GS regulates the content of Gln, which activates the TOR signalling pathway by promoting AKT and inhibiting AMPK. Furthermore, TOR regulates the fecundity of *N. lugens* probably by mediating Vg expression (Fig. 5G).

## Discussion

Targeting a gene via RNAi is often used to study its function.However, the reports on the global changes of mRNA and protein profile that occur after RNAi targeting of a specific gene have been limited^21^,^22^. For example, after knock down of ISWI in *Drosophila* SL2 cells, Bonaldi *et al.* studied the global changes of transcriptome and proteome using microarray and SILAC-MS technologies, and found that there is a very little correlation between mRNA and protein level^23^. In cultured male-like tissue cells of *D. melanogaster*, the expression level of many genes on the X chromosome decreased after RNAi targeting MSL complex; however, the gene expression on autosomes remained largely unchanged^24^. In our study, the combinative analyses of iTRAQ proteomic and DGE transcriptomic data after GS RNAi revealed 52 overlapping pathways, including the TOR pathway (Fig. 1). We identified 12 genes in which 5 were up-regulated and 7 were down-regulated at both mRNA and protein level (Supplementary Table 2). Similar to the previous report^23^, the fold of change in mRNA and protein levels was different.

Recently, accumulated evidences showed that the TOR pathway regulates protein synthesis and cell proliferation through integrating multiple upstream signals^25^. In *N. lugens*, the knockdown of TOR leads to decreased fecundity that is correlated with lower Vg expression (Fig. 4), implying that TOR regulates its fecundity probably by mediating Vg expression. However, TOR could also affect fecundity by other mechanisms. While in *A. aegypti*, TOR pathway is essential for the activation of Vg expression^26^,^27^, TOR-dependent activation of S6 kinase is a crucial step in the transduction of nutritional information during egg development of mosquitoes^28^. Moreover, in *Drosophila*, TOR regulates ovary size^29^, and is a determinant of follicle number by promoting cell survival^30^. In Apis mellifera, TOR governs diphenic development^31^. Both phenotypic and mechanistic studies are desired in order to explain roles of TOR in regulating fecundity of *N. lugens*.

TOR is a well known nutrient sensor in many organisms^8^,^32^. In multiple types of cancer cells, a positive correlation between Gln and TOR was observed^12^,^13^,^14^. Consistent with this, we found that Gln activates the TOR pathway via phosphorylating S6 K (Fig. 3). However, a different result was reported when Ba/F3 cell line was used^15^. Possible explanation is that the effect of Gln on TOR signalling may be cell type dependent^15^. Another possibility concerns a threshold effect of Gln concentration in cells. Since all experiments of this study were conducted in *vivo*, the findings may be more physiologically relevant.

Interestingly, we also found that Gln apparently promotes AKT activity, while inhibiting AMPK activity in *N. lugens* (Fig. 5G). This finding provides evidence that Gln regulates TOR not by a single pathway but by a network composed of growth (insulin) and energy pathways. However, it is still not clear how Gln stimulates AKT phosphorylation/activation. In mammalian systems, Gln stimulates synthesis and secretion of insulin-like growth factor 2 (IGF2) as well as IGF2-dependent AKT phosphorylation in beta cell^33^. In addition, Gln, Leu and Pro increase AKT phosphorylation in HepG2 liver cells^34^, presumably, through the inhibition of NF-κB. Also, how Gln inhibits AMPK is an open question. It is thus interesting and important to understand the mechanism underlying Gln-mediated AKT activation and AMPK inhibition.

## Methods

### Insect Rearing

A *N. lugens* laboratory strain that was originally obtained from Guangdong Academy of Agricultural Sciences (GDAAS; Guangdong, China) in September 2007 was used, and this strain was reared in a continuous laboratory culture on BPH-susceptible rice plants (Huang Hua Zhan, bought from GDAAS). The insects were maintained in the laboratory at 26 ± 2 °C with 80 ± 10% humidity and a light-dark cycle of L16:D8 h^20^.

### iTRAQ Analysis

To enrich the total proteins, two repetitions of the samples 72 h post-injection of dsGS were separately ground into powder in liquid nitrogen, homogenised in RIPA lysis buffer (Beyotime, China), and centrifuged at 12,000 rpm at 4 °C. The supernatant was precipitated in 10% trichloroacetic acid (TCA)/acetone, centrifuged at 12,000 rpm at 4 °C, and redissolved in dissolution buffer (0.5 M triethylammonium bicarbonate, 1% sodium deoxycholate) with 5 min heating at 90 °C and probe sonication. The protein concentration was measured with a Bradford assay protein assay kit (Bio-Rad, USA). The 4-plex iTRAQ Labeling, Strong cation exchange (SCX) and RP HPLC-MS/MS were performed by Fitgene Biological Technology Co. Ltd (FITGENE, Guangzhou, China). The peptides were identified by the Peptide Prophet algorithm^35^.

### DGE Analysis

The total RNA was extracted using the E.Z.N.A.® Total RNA Kit II (Omega) according to the manufacturer’s protocol. To obtain ideal gene expression information after RNAi, 48 hours post-injection of *dsGS* or *dsGFP*, the samples were used for transcriptome analysis. The genes differentially expressed between the two samples were identified using an algorithm as previously described^36^. The cDNA library was constructed and sequenced by the Beijing Genomics Institute (BGI, Shenzhen, China) on the Illumina sequencing platform (HiSeq™ 2000).

### Western Blotting

Western blotting analyses were performed using standard techniques. The proteins were separated on a 12% SDS-PAGE gel and transferred to PVDF membranes (0.4 μm, Millipore), and the membranes were immunoblotted with AKT, phospho-S473 AKT, phospho-pT183+T172 AMPK (1:2000, Abcam, UK), phospho-T389 S6 K (1:1000, CST, USA), GS, Vg and S6 K (1:5000) serum (prepared by our lab). IgG goat anti-mouse and goat anti-rabbit antibodies conjugated with HRP were used for secondary antibodies (1:5000, Abcam, UK), and the membranes were visualised by ECL (enhanced chemiluminescence).

### Quantitative Real-Time PCR Analysis

The primers used for real-time PCR are listed in Table 1. The synthesised first-strand cDNA was amplified by PCR in 10 μL reaction mixtures using a Light Cycler 480 system (Roche, USA), and the *β*-actin gene was used as an internal standard^37^. After the amplifications, a melting curve analysis was performed in triplicate, and the results were averaged. The quantitative variation was calculated using three independent biological samples by a relative quantitative method (2^−ΔΔCT^)^38^.

### RNA Interference and Sampling

The dsRNA of a target gene was produced using the T7 RiboMAX™ Express RNAi System (Promega, USA). After synthesis, the *dsGS* (439 bp), *dsTOR* (457 bp), *dsAKT* (447 bp) and *dsGFP* (688 bp) were quantified by ultramicro-spectrophotometers (NanoDrop 2000, Thermo) and were maintained at −80 °C until use. The sequence was verified by sequencing (Invitrogen Company, Shanghai, China). To deliver dsRNA into the body of BPHs, the dsRNA was added to the artificial diet^39^ or directly injected^40^. Before injection, the dsRNA and phenol red solution were mixed for observation. The purified dsRNA or ddH_2_O were slowly injected using 3.5 Drummond needles and the NARISHIGE IM-31 (Nikon, JAPAN).

### AKT and AMPK Activity Quantitative Test

AKT and AMPK quantitative enzymatic activity test kits (Genmed Scientifics, Inc.) were used to conduct to detect the activity. According to the manufacturer’s protocol steps, the absorbance was read at 340 nm. Finally, the AKT and AMPK active quantitative indexes were calculated using separate formulas^41^.

### Glutamine Synthetase Activity Assay

Glutamine synthetase (GS) was assayed using the method with a modification^42^. The assay medium included 230 μl of reaction mixture (100 mM Tris-HCl pH 7.4, 80 mM MgCl_2_, 20 mM cysteine, 20 mM sodium L-glutamate, 2 mM EGTA and 80 mM hydroxylamine hydrochloride). The reaction was started with the addition of 100 μl of enzyme extract and 100 μl 40 mM ATP, incubated for 30 min at 37 °C, stopped by the addition of 143 μl of a chromogenic agent (0.37 M FeCl_3_, 0.2 M TCA and 0.6 M HCl) and placed at room temperature after a moment. After centrifugation, the absorbance of the supernatant was read at 540 nm against a reagent blank.

### Statistical analysis

The results are expressed as the means±S.E.M. SPSS 13.0 software was used to perform t-tests to identify significant differences at a 95% or 99% confidence level.

## Additional Information

**How to cite this article**: Zhai, Y. *et al.* Activation of the TOR Signalling Pathway by Glutamine Regulates Insect Fecundity. *Sci. Rep.* **5**, 10694; doi: 10.1038/srep10694 (2015).

## Supplementary Material

Supplementary Information

## Figures and Tables

**Table 1:** The High Confidence Quantitative Proteome from GS/GFP RNAi: Size and Features.

Identified nonredundant peptides	77,809
Identified nonredundant proteins	1,151
Quantifiable Proteins (with unique peptides >0 + non unique >1)	958
Differential expressed proteins (*p*-value < 0.05, ratio ≥1.2 or ≤0.8 )	124
Up-regulated Proteins	49
Down-regulated Proteins	75
Minimal peptide length (aa)	5
False positive rate (FPR) proteins	2%
