# Supplementary material for: Activation of the TOR Signalling Pathway by Glutamine Regulates Insect Fecundity
Source: Sci Rep. 2015 May 29;5:10694. doi: 10.1038/srep10694 (PMC4448656; doi:10.1038/srep10694)
Supplement: Supplementary Information [file srep10694-s1.pdf]

Activation of the TOR Signalling Pathway by Glutamine Regulates Insect Fecundity

Yifan Zhai[1,2], Zhongxiang Sun[1], Jianqing Zhang[1], Kui Kang[1], Jie Chen[1], Wenqing Zhang[1*]

[1]State Key Laboratory of Biocontrol and School of Life Sciences, Sun Yat-sen University, Guangzhou, 510275, China, [2]Institute of Plant Protection, Shandong Academy of Agricultural Sciences, Jinan 250100, China.

[*] Corresponding author:

E-mail: lsszwq@mail.sysu.edu.cn

Tel: +86 20 39332963,

Fax: +86 20 39943515.

Supplementary figures

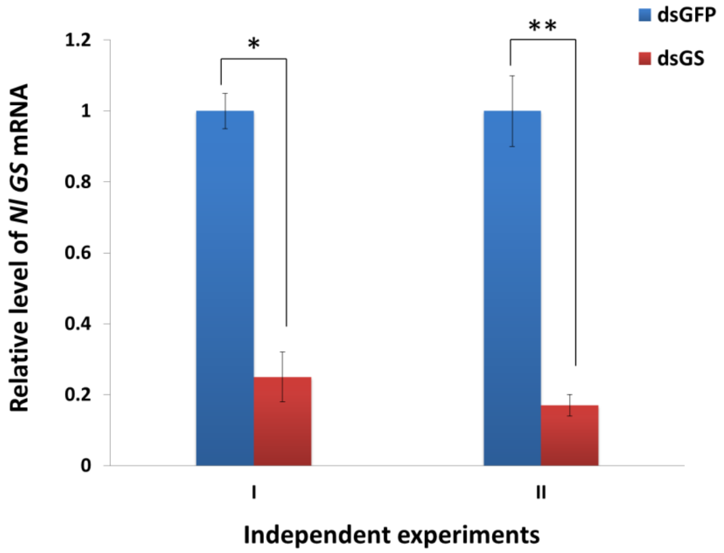

Figure S1. The mRNA expression of the GS gene in the 2 samples for iTRAQ. Data represent mean values ± S.E.M, **p*<0.05, ***p*<0.01.

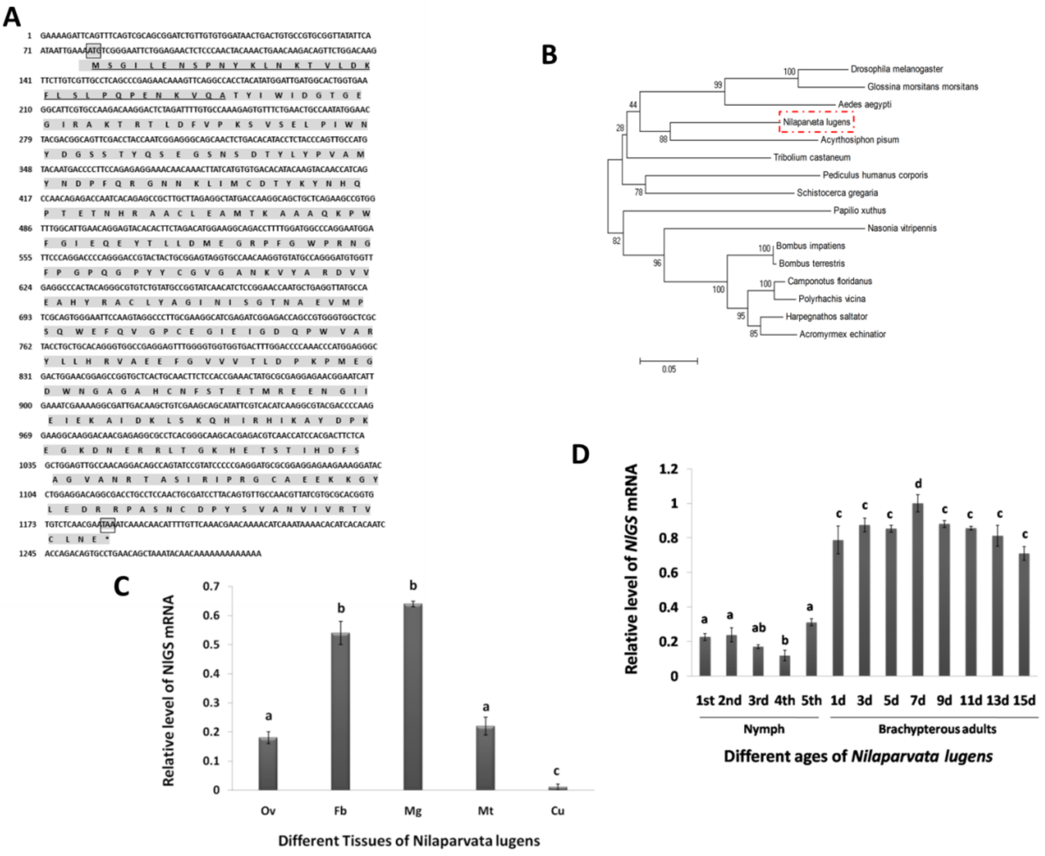

Figure S2. The sequences, phylogenetic tree and expression of GS gene.

(A) Nucleotide and amino acid sequences of the full-length GS (GenBank accession No: KC445137.1);

(B) Phylogenetic trees for some known GS genes;

(C) Different Tissues expression of GS mRNA by qRT-PCR;

(D) Developmental expression of GS mRNA. Data represent mean values ±S.E.M (n=3), and those on the same column followed by the different letters means significant difference (*p*=0.05, Duncan’s multiple range test).

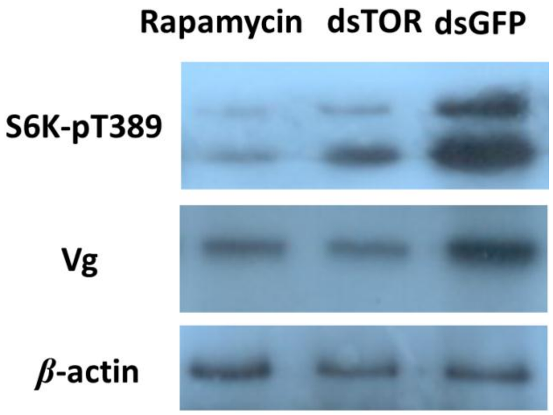

Figure S3. The S6K phosphorylation and Vg protein levels in different treatments. First day brachypterous female adults were injected with ds*GFP* (250ng), ds*TOR* (250ng) or rapamycin (10 mM). Samples were used 48h post-injection.

Supplementary Table1. Proteins identified by iTRAQ and RP HPLC-MS/MS

No.	Accession No.	Unigenes No.	iTRAQ Label		Protein name	Partial Peptides identified	GSi / GFPi Regulated
			114:115	116:117			
**a1**	gi|237874159	NLU022777.1	1.753880978	1.706081986	ATP citrate lyase *[Acyrthosiphon pisum]*	AALYKLYTDLHFTYLEINPLVVTADSIYILDLAAKLDATADFICRQQWGEIDYPPPFGRDAFPEEAYIADLDAKSGASLKLTILNKKGRIWTMV AGGGASVIYSDTICDYGGASELANYGEYSGAPSEQQTYEYAKTILSLMTQEKHPQGKVLITGGGIANFTNVAATFKGIVTALTEFQGKLLEHNI SIYVRRAGPNYQEGLRIIRDVGKTLRIPIYVFGPETHMTAIVGMALGKKPIPKETEMEFATANFLLPGGQDKGSSSEKSSQSSSVSPSGDVGSSC PMPASSSSKTMFTNKSKAIVWGMQTRAVQG	Up
**a2**	gi|157133341	NLU001572.2	1.19124198	1.235947013	Citrate synthase *[Aedes aegypti]*	FAKAYSSGVPKAKYWEHCYEDSMDLIAKLPVIAALIFRNTYKDGKSVGTIDPKLDWSANFVNMLGVNNTPEFTELMRLYLTIHSDHEGGNVS AHATHLVGSALSDPYLSFAAGMNGLAGPLHGLANQEVLVWLTKLQQQLGADPTDEHVKEFIWKTLKGGQVVPGYGHAVLRKTDPRYTCQR EFALKHLPNDPLFKLVNQIFKTVPPILTELGKVKNPWPNVDAHSGVLLQYYGLKEMNYYTVLFGISRALGVLSSLIWDRALGLPIERPKSFSTE KLIKDYSK.	Up
**a3**	gi|193659676	NLU022227.1	1.137627006	1.330453992	Phenoloxidase subunit A3-like *[Acyrthosiphon pisum]*	SIEVNSMNSPKNQFSTFWQQNDVNLSRGMDFAPRGNVFARFTHLQHRPFEIKIQVANNGQARQGTVRIFLAPTMDERGVPFLFNDQRRLMIE LDRFVHNFQPKNNTVTRRSNQSSLIIPFERTFRDLNVANNANLAEFNYCGCGWPEHMLIPKGTPEGFPCTLFVMVSNYAEDRVDVPNRQPTSC EGAMIFCGMKDSRYPDKRPMGYPFDRLPRTGVDTMRDFLLPNMFTQDVVIRFRGTAAPPKRN.	Up
**a4**	gi|641672799	NLU001692.2	1.380383968	2.128139019	Trifunctional purine biosynthetic protein adenosine-3-like *[Acyrthosiphon pisum]*	GENIIIEEVLTGVEVSVLAFTDGTEVRVMPPAQDHKRALDGDQGGMTGGMGAYCPCPYIDQQGLEMVEKTVLQPVIDKLRQDGSPFVGPCC YVFATLHRKQLFSSPYIDLKDIVCRRLSSDVRTCFFPAKAGKSTLSGRFLKIFENVFGQLCHMVHCVQEGVLYAGLMLTKDGPKVLEFNCRFG DPETEVLLPLLDTDLCDIMKACCTKQLKNIDIQWKKNLSAVTVIMASRGYPESSSKGDVIQGLSEAESLDNTLVFHCGTRLADNDVISTNGGR VLAVVALANQLSAAAAKANTGCAKIRFPGAQ	Up
**a5**	gi|193582492	NLU028445.1	1.29419601	1.19124198	Histone H2B *[Acyrthosiphon pisum]*	MPPKTSGKAAKKAGKAQKNIAKGDKKKKRKRKESYAVYIYKVLKQVHPDTGISSKAMSIMNSFVNDIFERIAAEASRLAHYNKRSTITSREI QTAVRLLLPGELAKHAVSEGTKAVTKYTSSK.	Up
**a6**	gi|641675779	NLU010113.1	2.312064886	1.659587026	Lysosomal alpha-mannosidase-like isoform X2 *[Acyrthosiphon pisum]*	KQSNGSNVNLLYSTPSCYVKAVNEDNLSFTTKKDDFFPYASDPNSFWTGYFTSRPTIKYFERKGNNFLQICKQLYALADLGPEDRGDLNALRS AMGVMQHHDAITGTEKQLVAFDYARILSQGMAECEIVVDAALRKIIAPKTADTEDQPKLDDEFKTVTCPLLNVSQCATTEENKSFVVTVYNP LSKPVTHYVRLPVNDNANYTVLCPMGKKQVTQLVPISEAVKRIPGRASKAEFELVFRAKNLPPLGFRSYYVSQDAKLDGEEHDYTGGQVRR EPTENIVLGDHMGLSLTLNENGELSSATFGEKTIKLSQGIYYYSGA	Up

**a7**	gi|646714120	NLU028533.1	1.127197981	1.51356101	Inositol-3-phosphate synthase 1-B *[Zootermopsis vadensis]*	GKDVCIPMADMLPMLRPEDIVLDGWDISGLNLAESMERAQVLDYNLQVQLAPLMKDMKPRPSIYFPDFIAANQSERADNVLTGTKWEQLE AIRKDIRDMKEKQNVDQVIVLWTANTERFAEIIEGVNDTADNLIAAIKRNEAEISPSTIFAVASILEGSTYINGSPQNTFVPGCMELAERYRVFIA GDDFKSGQTKLKSVLVDFLVNAGIKPVSIVSYNHLGNNDGRNLSAPSQFRSKEISKSNVVDDMVDSNSILYKPGEKPDHCVVIKYVPYVGDS KRALDEYTSELMLGGHNTISIHNTCEDSLLAAPIILDLVILAELCSRIQFKAENQQTYHQFHPVLSVLSYLCKAPLVPSGTPVVNALFKQRACIE NIMRACLSLPPDNHMTLEHKLPKSLFSTNVNTDKIVDKISNTCFITKPEKPQQKVVA.	Up
**a8**	gi|112983802	NLU019205.1	1.116863012	1.14815402	Mn superoxide dismutase *[Bombyx mori]*	LIRVAGASRQKHTLPELPYEYNALEPVISREIMSLHHSKHHATYINNLNVAEEKLAQAQAKGDIDTIINLAPALKFNGGGHINHSIFWHNLSPN GGKPSDVLTKAVEKDFGSWDNIKNQLSTASVAVQGSGWGWLGYNKQMKKLQIATCQNQDPLQATTGLVPLFGIDVWEHAYYLQYKNVRAD YVKAIFDVA	Up
**a9**	gi|339765122	NLU011146.1	1.406048059	1.19124198	Cytochrome P450 CYP6ER1 *[Nilaparvata lugens]*	LCSLVPRGAEGKTLKIKEITNNYGLNVIASTAVGIDHNSFEKENPLADAALKVTDPDDLMQGLRFILSFVSPKIAKFFNMRFTPKGVSDFYIDM VDKIVNYRKSHNVVRKDFMQVLLNLNEEIEKSKESDGREPLSLDEMASQTFLFILAGHESTSASLCFLLYELAVNQEMQQKLYDEIKAVDGDI TYETIKELEYMDMIFNEMLRKYPGPVLIRLCVKDFILPNGFLIRKGTQVMIPVYALQKDPKYFPQPDKFEPERFSKRAPIHEIIPFSFIPFGEGPRY CIGKRFGIASVKLGLIHILSKFKILPASDTKIPLEIEKKTFVLNPYKDLTLKLIARDT.	Up
**a10**	gi|641665969	NLU002171.1	1.432188034	1.472311974	AP-1 complex subunit beta-1 *[Acyrthosiphon pisum]*	NLDTLDEPEARASMIWIIGEYAERIDNADELLESFLEGFHDENTQVQLQLLTAIVKLFLKRPSDTQELVQQTVAARPDNSAHTHIPGRHVMVLS LATQDSDNPDLRDRGFIYWRLLSTDPAAAKEVVLADKPLISEETDLLEPTLLDELICHISSLASVYHKPPTAFVEGRAGAGLRRSLPNRAGSQA GSRARPTVSAYVSRACLWFFHTYSMYPYRAGLTARMRSENVRETAAEASVIPTQESLIGDLLSMDISAPTVISTPTPAVDLLGGGLDTLLGGDV GTTAPPINQSTTGLLGDIFDFTPTPTMYMVPKIMWLPADKGKG	Up
**a11**	gi|91084795	NLU027896.1	1.235947013	1.235947013	Lamin Dm0 *[Tribolium castaneum]*	LELDRSRFMKEYTALEEEMNKLREEMSRQLQEYQDLMDIKVALDMEIAAYRKLLEGEEARLNITPVQSPGRPSPGRRTPAGRGGKRKRTLME ESSEEASSSSYTVKASSSGDIEIAEICPQGKFVKLHNKSGKEVSIGGWQLIRIVNDATTTFKFHRTVKVEPDGFVTVWSADANQQHEPPANIVM KGQTWIVGEKIATTVYNSNGEEVATAEHVRQQSSQSSSSLRKRVFPQSSFRSSSEQELYHQQPPEQQHVRSVAATLVQRCGGRRKLAELAVDG SLFRLLCSAIAMDDVAAATDAATATVIYCEYL.	Up
**a12**	gi|227430130	NLU021826.1	2.249054909	1.235947013	Cytochrome P450 CYP6CS1 protein *[Nilaparvata lugens]*	GKSAPTVTFNPALFLNSPKDGLYIRFVPRTDFQVRLRIALESFTMSSLLIGGWGWIISTLLTTTLLFLFIFLKRKLNFWENLGIPTIPGSFPFGSVSR LATFQLSTAELFKEFYDRGRGKDYLGYYTLFKRSLLVRDTELIKNILVKDFSSFLDRGVYHNLENDPLSGHLFSIEGDYWRSLRNKLSPTFTSG KMKTMYNTVKDLSEDLKKYAMQKASTDGNVEMKDLTARFGLDVISNVALGLEGNNFNDENSEMKHMTEMMLKPSFFQYTKTVMSMVCK DFLIHLRIRLTPMPVENFFMKLVRKNIEYRERMKITRKDFMQLLIDMKESSFTNNKDRHNEKLQEGEKLTLTLEEIAAQSFVFVLAGFETSSVT LGFMFYELASYP	Up
**a13**	gi|91085003	NLU001599.2	1.51356101	1.330453992	Glycogen [starch]	HLCHSFTTVSEITGYEAEHLLKRKADIITPNGLNVIKFSALHEFQNLHALAKDKIHEFVRGHFYGHYDFDLDKTLYFFTAGRYEFTNKGADIFI	Up

					synthase *[Tribolium castaneum]*	EALARLNHYLKTSRQDVTVVAFMIFPARTNNFNVESLRGHAVTKSLRDAINEIQSKMSKRIYEICLSGRMPDNEELMTKDDKIKIKRCLYALQRNGLPPVTTHNVVDDWNDPVVFHPEFLSSTNPLFGLDYEEFVRGCHLGVFPSYYEPWGYTPAECTVMGIPSITTNLSGFGCFMQEHIADPMSYGIYIVDRRYIGLEDSVKQLAQYMFDFCRLNRRQRIIQRNRTERLSDLLDWRNLGIFYRQARHKALQKVYKDVESFEEEYEVRRNSFSFPRPISEPPSPSASRHTTPASSVHGSEDEDEVDDEKEVT.	
**a14**	gi|91092464	NLU006709.1	1.16949904	1.158776999	Glycine dehydrogenase (decarboxylating), mitochondrial *[Tribolium castaneum]*	ITYPSTFGVFEETIAEICRMAHSYGGQVYMDGANMNAQVGLCRPGDYGSDVSHLNLHKTFAIPHGGGGPGMGPIVVKEHLASFLPSHPVVELVDPQRPNSFGAVSAGPFGSSCILPISWAYIKMMGAEGLRRATQVAILNANYMSKRLEGHYKTLFQSPESGLVAHEFILDIRDFKKTANVEAADIAKRLKKAGQRGGGGHCQAADGLRLPCSHHVLAAIREEIAMIEEGKMDKHLNPLKLAPHTQQQVLSDKWDRPYSRELAAFPREFVRADNKLWPTVGRIDDIYGDKHLVCTCPPILPSYNY.	Up
**a15**	gi|328700272	NLU027606.1	1.445440054	1.116863012	Gastric triacylglycerol lipase-like *[Acyrthosiphon pisum]*	LYYIGHSMGTTIFWVLCSMRPEYNSKIKMMFSLAPIGFMSNARSPIRYFAPYAKDIELISRWFGDGEFLPRNALINFVMKYGCEISALEAKICENNLFIICGHDPEQFEKELLPIIFGHTPAGTSTKALAHYAQLIQSGYFRAFDYGLPGNMIEYGEITPPSYNLSLITAPVSLHFSDNDLLANTKDVKKLAMALPNLIGSFRVAFPQFNHLDFLYSKDINSLLNENIMNMIHKSESGITPRSNMDPEEIIIPNEDEDPVFNDISSLLKHKAYSIGKETLDNVMDLKMMTERKLKMIKEKTRMTIERNLKSLKDLLMLKLKKYSTGDSYGENKQIEFRNDAEKYNDLSS.	Up
**a16**	gi|193580067	NLU010922.1	1.28233099	1.258924961	Lysosomal alpha-mannosidase-like *[Acyrthosiphon pisum]*	QEYNMTFNATEDVVVDNGVLQLIFNGSTGLLEYVTKDNETWLFRQNFYYYEASKGYNYNSDNRASGAYIFRPAKETTYLISEQVQISVFKGKEVTEVHQVFDTWLSQIVRIYKNQDQIEFQWLVGPIPVEKWTGKEVITKYKTTLQTNGELWTDSNGRRMIKRKRNYRSSWNLTLTEPVASNYYPITSAAAIKDDFRQNFYYYEASKGYNYNSDNRASGAYIFRPAKETTYLISEQVQISVFKGKEVTEVHQVFDTWLSQIVRVYKNQDQIEFQWLVGPIPVEKWTGKEVITKYKTTLQTNGELWTDSNGRRMIKRKRNYRSSWNLTLTEPVASNYYPITS	Up
**a17**	gi|642926044	NLU027866.1	1.137627006	1.106624007	Acetyl-CoA carboxylase isoform X3 *[Tribolium castaneum]*	MKMIQRRNSKRFILSELKTGEEEEEGEEVTATAQTPSEIPTDPISFLDRPPTHHEILKWKSIRSLRAQISPSMSQGTVIHKGIMDKDFTVGTPEEFVRRFKGTRVINKVLIANNGIAAVKCMRSVRRWSYEMFKNERAIRFVVMVTPEDLKANAEYIKMADHYVPVPGGTNNNNYANVELVVDIAVRTQAQAVWAGWGHASEYPKLPELLHKNNIAFIGPPEKAMWALGDKIASSIVAQTAEIPTLPWSGSELRAQYSGKKIKISSELYKKGCVASVDEGLASAQKIGFPVMIKASEGGGGKGIRKVESSEEFPNLFRQVEKRLDSSQDHVFSNDDVAETRIDWSECHHLKTHGLD.	Up
**a18**	gi|156542034	NLU025371.1	1.137627006	1.247382998	Peroxiredoxin 1 *[Nasonia vitripennis]*	MARVQETAPAFDGTAVIDKEFKEIKMSDYKGKYVVLFFYPLDFTFVCPTEIIAFSDRAQEFEKINCQVIAASCDSHFTHLAWINTARQNGGLGEMNIPLLADKSGAIARAYGIYDEKTGVPFRGLFIIDGKQKIRHITVNDLPVGRSVDEALRVVQAFQFTDEHGEVCPANWKPGKKTMKPDPKGAKEYFQSS.	Up
**a19**	gi|642929299	NLU004401.1	1.445440054	1.235947013	DnaJ homolog subfamily C member 13 *[Tribolium castaneum]*	TKYIVGMLERCIDRKERDRFLQFLDKLMHDKKNVKAVIDANGVQIFVDLMTLAHLHTSRAVAPTQTNVIEAGKDMVREAEKEWYYGTGGEKKGPVSFLKMKELWQRGEINGQTKCWAQKMERWMPLQAIPQLKWCLAAKSEPVMNESELASLILSMLIKMCEFYKSRDSNGAVIWPMPRIKRELSDPNCLPHIVQLLLTFDPVLVEKVATLLCHIAEDNALVSKLYDTGVFYFIMMYRGSNLLPIARFLKLTHTKQAFKLSDEDHSSELMQRSV	Up

						LGQLLPEAMIYYLENHGPNQFTKIFLGEFDTPEAIWNSEMRRFLAEKIAYHIADFTPK	
**a20**	gi|641663105	NLU027219.1	1.127197981	1.16949904	Annexin B9-like isoform X2 *[Acyrthosiphon pisum]*	EVLRKAMKGFGTDEKAIINVLANRTNAQRQEIAVKFKTLYGKDLIKDLKSELSGNFEDLVVALMTPISSLLARDLNKAVSCIGTEEETIIEILCTATNHEIYAIRTAYQSMYGTSLEDDMASDTSGSFRRLLVSLCQAARDENYIVDMAAAQNDAQKLLRAGELRLGTDESSFNAILCSRSYPQLSQIFLEYQRLTGHDFSKAIENEFSGDIKDGLLAIVKTVRDRYAFFAEQLYNSMKGFGTKDRALQRIVAVRSEIDMVEIKRAFTAKYGKSLEEFIHDDTSGDYKKCLLALVSDV.	Up
**a21**	gi|219551908	NLU015814.2	1.976969957	1.499685049	Farnesoic acid O-methyltransferase *[Nilaparvata lugens]*	GRAKHDSGALIPGKVVPSHGVCYVPWGGEEHGIAEYEVLTGCEVTWMPSASGQVPSGALEAGQTEDGETLYIGRATHEGASAVGKDSEQCPGDSTVLLKDVSGNRISHLDEGGVHNLEMSALVSIGPSLQLTVESGTARTSAYLLVHGQGSHRVTGSLKSLVWNHLEPRSRTSSWGDKSPLVRVVGTLGNRLSHVAKNSDIETGQRFPVQGTHGVCYIPYGGAEIPYNDYEVLVAK.	Up
**a22**	gi|642922839	NLU001598.1	1.213389039	1.485936046	Isocitrate dehydrogenase [NAD] subunit gamma, mitochondrial-like isoform X2 *[Tribolium castaneum]*	HQDIDIIIIRQNTEGEYAMLEHESVYGVVESMKIVTVENSERVSRFAFEYAKKAGRKKVTTIHKANIMKLSDGLFLETSRKVAKEYPEIEHNDMIIDNCCMQLVSNPHQFDVMIMTNLYGTIVSNVVCGLVGGAGLLSGKNYGDHYAIFEPGTRNTGTAIAGKNIANPIAMLNAGVDMLEHLGHFVHADILQNAIDKTVNEDRIHTPDLGGTAASTDVVQNIIRHVQTATKNMKWKIVALGFASGFKLFPKGEKTALFTLDLQITISLYFLIYLGPYSYSKCEPKLSS.	Up
**a23**	gi|193636528	NLU003053.1	1.137627006	1.247382998	Protein takeout-like [Acyrthosiphon pisum]	MHSSILQEKTNLAKRNAVWKFAHERVEMIGDYSVNGKILVLPIKGEGKCNITITDVKVDFEMNWNLIKKDNGKEYINFTTTDLKLSNGRAYFKLENLFGGDRTLGDNMNQFLNENWKEVTQDVGPALGKAMGDVFTLLVSNLNNVVSYDNIYPPS.	Up
**a24**	gi|646711374	NLU011985.1	1.258924961	1.158776999	Phosphohistidine inorganic pyrophosphate phosphatase *[Zootermopsis vadensis]*	MSSCPLRITKMAKVEWQSKPVKGLLIDISGGLKTGIEAIPGSVEGLRLLYESGIPFRIVTNESQCNVSQLLRMLQRMGFHLKADDIFSPIPAVVELLRKENLRPHLIVNPSVESEFDEFKTEEEPNCVVMADAQEHFNYDSLNKVFRLLIDMEKPQIFTLGTGKYYEGDDGLLHLDVGPFTKAMEYATGLKARIVGKPSSDFFLSALDDLGIKPEEAVMIGDDVVSDVGGAQNSGMRGILVRTGKYRTKDEQHVIKPDAIFNNFNDAIHAILNI.	Up
**a25**	gi|170053486	NLU017753.1	1.202263951	1.393157005	Histone H3c *[Culex quinquefasciatus]*	MYTQEGKPLQEEKYVPSIHTLPSFRIMNMNLKSLTQSKPATGGVKKPHRYRPGTVALREIRRYQKSTELLIRKLPFQRLVREIAQDFKTDLRFQSSAVMALQEASEAYLVGLFQDTNLCAIHAKRVPIMLKDIQLARRIR.	Up
**a26**	gi|645011944	NLU026876.1	8.016780853	4.965922832	Protein dopey-1 homolog isoform X3 *[Nasonia vitripennis]*	LWDCLASNAAVRLPAILFALAHYDKRLSTEDQLHVMGTNVDVMVNGLCACVQDTSVLVQRSALDFLLVGFPMHNSQLLRCDMVRLVTAALSTILRRDMSLNRRLYAWLLGSEINIALLSPEHPLVKRSKTADSITSNISTSQYFELYSREMLVLAIKTLLDQATGQTPHDLRPYRLLVSLLDKPDIGPVILDDFLFEVFRKGSCTSKGDITWRCLVKNCSVSIKTNSSASIIREINGSHTHDSLAISSIETDEESLLLSPSNVGKEINFNDSNICDKSQTTHESALELENLQ	Up
**a27**	gi|91092172	NLU007777.1	1.55596602	1.629295945	Probable	RFDVFCHALESFTAVPFNERGPCPPGPGPQNPILRPAYQGQNPVSDVWARFALQVIAKYFKRPNMHLASTMAGVGFGNAGVHLCHGLSYPIS	Up

					hydroxyacid-oxoacid transhydrogenase, mitochondrial *[Tribolium castaneum]*	GNVKTFQPEGYSDDHAIIPHGLSVVMSAPAVFGFTGPACPQRHIGAAECLGKDVRNVKRDDAGAVLADTVREFMHVMKVENGLSALGYKK SDIPGLVQGTLPQAKALELKRELRKTEWEAFVVDMDAKLQKVDETFQEKENELKEFYCDLERKLHLTAVFRMLDSIEDPEIKISLNKEKLSRV KESSREELPQRFFEEIRVGDVGWLAERRAILGDVPLHELLSGCDIALPRPVEVKRSAQLEERILKLKQEQHEREYRDMTKNVDNVRVRHPEDT IAFQSNIAHFSSSY.	
**a28**	gi|157104119	NLU024668.1	51.05049896	13.55189037	Aromatic amino acid decarboxylase *[Aedes aegypti]*	LEVVMLDWLGKILGLPNEFLACSGGKAGGVIQGTASEATLVALLGAKARAVRKAKEQHPDWTDAVINSKLIAYASEQAHSSVERAGLLGGVT FRNLKVDSHYRLHGDTVDEAIKKDLKEGLIPFYVVATLGTTSVCSFDLVTEIGPVCRKHDLWLHIDAAYAGSAFICPEYRYLMEGVELADSFN FNPHKWMLVTFDCSAMWLKDPTDVIDAFNVDPLYLKHEQQGSAPDYRHWQIPLGRRFRSLKLWFVMRLYGVKNLQAHIRKQIGFAHQFEA YVNEDERFELVGDVLMGLVCFRLKGSNELNEKLLKRINGNGKIHLVPSKIDGLYFLRLAICSRYTESTDIEFSWTEIKSLANDVLVEEK.	Up
**a29**	gi|157133746	NLU024891.1	1.258924961	1.180320978	Saposin *[Aedes aegypti]*	RFVNTFTEDIVNAVMADFNEKEVCAYLKMCSAEVMSTDVVTFDPHATNKPATETVDTKKPATETVATKKPETETVATKKPETVEKTTPVNIET NEVHDDVTVGDNKQKCVLCEFVLQRIDNELKDKKTEDEIKHAIHTACSHMPHTVVNQCNQFINQYADLIFDLLADSLEPKQICTKIGLCKND TVVEEVVEVKSEEMKRDASFDETSAKIVYSCLGAVGVIAVIVLVATVSYRTRCLACEALLGSLQAISEDPIVRSSLNGELIDFCDQLPPKMHSPC KDVVMQLAPQMETILQEIPLGPIVCYRAKNICRPYHVSGIHTHVMAAGCEKGPDFWCKTTSNAVLCGTSVYVGSMVNYKGEVCDSLASVHI HCFGAHSALYVAAPMELAVAPFFDCDFLGVVPPATTHQLAAIHTV.	Up
**a30**	gi|157114308	NLU021306.1	1.158776999	1.14815402	Tryptophanyl-tRNA synthetase *[Aedes aegypti]*	MLFYFQKEEKWETRIFSGVQPTGAIHIGNYFGAIEKWVSLQDEGNRTIYSIVDLHSITLPQDPMELRANILLMTATLIACGIDPKKSIIFQQSKCS NNIILYCKRPLPILVTSTHVPVGEDQEQHMQIAQTLARQFNNRFGVTFPIPQTIMASDERNRVRSLREPQRKMSKSHADAKSRIQLTDTPEVIVE KVTNLLIIHTQLLFYENDVATLRTELDKDSAIRFVE.	Up
**a31**	gi|568255707	NLU028274.1	1.106624007	1.14815402	ADP ribosylation factor 102F *[Anopheles darlingi]*	MPHAWVEARVRQRGVGESESKIKTLYFMCERMTERCGSGKVSKYSFRDRIRTAKIMGLTISSVFTRLFGKKQIRILMVGLDSVGKTTILYKLK LGEIVTTIPTIGFNVETVEYKNICFTVWDVGGQNKIRPLWRHYFQNTQGLIFVVDSSNRERIDEAAEELQYMMQEYELRDAVLLIFANKQDVP NAMTTSELGDKLGLNQIRNNWYIQATCATQGKGLYEGLDWLSNQLAK.	Up
**a32**	gi|112982830	NLU021877.1	1.753880978	1.55596602	cuticular protein glycine-rich 4 precursor *[Bombyx mori]*	AAKTEDKAARDKRSFFHGLGYDSNYGSTFGYGSGYQNLGGLGYSGYSSPSVYSSPSSYSYSTPAVSSYGYTSPAVSYANAGYRYASPAYSYAA PAFSAASYAAPVYSAASYAAPAYSAASYAPAVSSYGYSTPQVRYSSAPAVSYSSFTSVPSVKYAAAPAVSYSAAPAVSYSAAPAVSYSAAPALSY SAVAPAVKYSAAPAVSYSAAPALSYSAVAPAVKYSAAPAVSYTAPISYTAPAVSYTPAVPTTVAVNKVVNVPKVVSVPEVVKVNKVINEPQVVT VNKVVSQPSYNLGYSGYTGGLAGYGAGYGTYGSGLSTYGAGYTPYSTGYSTYGAGYTPYSTGYSTYGAGYTPYSTGYSTHGSGFGPYSTG FGTG	Up
**a33**	gi|328719143	NLU019746.1	2.208004951	2.312064886	Actin-interacting protein 1 isoform X2	KLWDVETKSVISQFTMGTQVEDQQVSCLWQGDYLLTVSLSGFISYLDVNNPDKPIRVIKGHNKPITVLTLSPDRKRIFTGSHDGYVTSWDAKT GETERVMGTGHGNQINGMRTVKNFLYSCGIDDSLKQVNLDSGWYSGVDIKLSAQPRGMDLKDDLVVVATVKELTVIKQDRKISGFPITYEPS	Up

					[Acyrthosiphon pisum]	SVSINPGDDSYLAVGGDDSKIHIYAMSGTVISPHKELQHLGPVTDVAFSPDGKYLVACDANRKVILYTLPDFQLAHNKEWGFHSARVNCIAWS PNSQQVASGSLDTTIIIWSVAQPAKHTIIKNAHPQSQITRLAWIDDETLTSVGQDCNTKIWNITPI.	
**a34**	gi|328717024	NLU017432.3	51.05049896	63.09572983	3-phosphoinositide-depe ndent protein kinase *[Acyrthosiphon pisum]*	MASSRFYAAEILVALEHLKRLNIVHRDLKPENILLDENMHILITDFGSSKIMTDHNDTNDEDDRSRARKNSFVGTAQYVSPELLTAKTASASSD LWALGCIIYQMVYGLPPFRSGSEFLIFKQIMNLNYEFPDGFCPVAKDLIEKLLIMNLNYEFPDGFCPVAKDLIEKLLVLEPSQRLGASDEVGYPSI RQHNFFQGIEFDTLQNQTPPLIYPYHSSTSDNHDLRSQYRVPDNLEPGLDDKQMTRLLGLELHESPKKQSLLTGLSADEVSRRLKQQEATNQF HRFVDGNLILKQGLVDKKKGLFARRRMLLLTTGPHLYYVDPIQMQLKGKIPWSNELSVEGKSFKAFHVHTPNRIYYLEDPEYLANEWCDAIN ELQQQHQT.	Up
**a35**	gi|328779619	NLU002669.1	1.116863012	1.127197981	Glutamate decarboxylase-like isoformX1 *[Apis mellifera]*	MENGERVRSKEEKREGNEEENYSKKGFEIFQKDNLDPNYSYGPFHKILKLNYMKPYSYATVGSILTDLLPHNESAFPVSKEFLQKVVDILMDF IKETNDRNCKVLDFHHPDKMRKLLDLDVPDKGVELQQLIEDCAKTLKYQVRTGQKSDLRIVKCDPRCDLSSIFVLFTELSKIIV.	Up
**a36**	gi|292606975	NLU001823.1	1.16949904	1.158776999	Vitellogenin receptor *[Nilaparvata lugens]*	SGSGLLEIVSTNLKQVSDIFVDSFHAHIYWVDSQTRKVERASFDGSNRQEVFTSPAVPTDITLFEDYMYVLVQADSASENIDMIETGNVWRCG LYGAAYQKCELFRIHPKHFTVPYHFEIMHPGLQLKGHNDCRNSTDCQSAGGMCLLRNHKLRPSAVCVCADGTRMRRNSICEAPSATIFDYDD VTYLTSNSPVQTSSLGSAFWMFLAFIFVVVPVVGILWFVYRGGPPSVPLSPPQWMPGFCARRFPFHTIRFNSKFGNMDADDTIPAYSDFQFHPC QLNPGEHQYENPIAAMQAEQNSGAAITIKTMNEIDIQMGEEKNGWKAGGGEGGDDSDSSTVEMPEVKLLNL.	Up
**a37**	gi|557756950	NLU022644.1	1.458814025	1.406048059	Phosphatidylinositol transfer protein beta isoform-like isoform X2 *[Musca domestica]*	MLIKEYRVVLPLTVEEYQVGQLFSVAEASKNETGGGEGVEVLKNEPFDNHPLLGGRYSKGQYTYKIYHLASKVPAIIRLLVPKGALEIHEEAW NAYPYCKTVITNPGYMKENFSIVIESYHIADAGDKDNVHELPPEKLKQRDVVMIDIANDPVSQGDYKKDEDPTTFSSEKTGRGPLTGKWMNQ VTPVMTCYKLVTAEFKWFGFQSRVESFIQKSERRLFTTFHRQVFCWLDRWHGLTMDDIRAIEDKTREELDKQRNVGEVRGMKAD.	Up
**a38**	gi|187179329	NLU017398.1	1.224616051	1.318256974	Twinstar *[Acyrthosiphon pisum]*	MASGVTVADACKKVYEEIKKDKKHRYVVFHIKDEKQIDIEVIGERNSTYDLFLEDLQKAGPQECRYGLFDFEYTHQCQGTSESSKKQKLFLL CWCPDTAKVKKKMVYSSSYDALKKSLVGVHKAFQATDHSEASQEVIEEKLRSTDRQ	Up
**a39**	gi|641647652	NLU021078.1	1.923092008	10.37528038	Zinc transporter 2-like isoform X2 *[Acyrthosiphon pisum]*	LQTDPTSPTETITLDTEDHCHKMRQDSVDKVARRKLIIASILCVFFMIAEVIGGVMSNSLAIATDAAHLLTDFASFMISLFALWVAARKPTRQLL FGWHRAEVIGALCSVLTIWVVTGILVYVAVERIIHQQYEIDSTIMLVTSAIGVLVNLIMGCTLHQHSHGHGGAEEHRGSNSINVRAAFIHVLGD FIQSTGVFIASILIYFNGPSWYIIDPICTFLFSILVMITTFTILKDTMLVLMEGMQRGVEYGDVLDTLLSIQHVEKVHNLRIWALSLDKTALAAHI VITICCLWKLPLTERCGGYVPSSPLSIVSSKQNELDKEQIKMLEAYERREECFTLLFSLSSAGKIVEPLAIVWIETENPIQIDTAQHISIDLTSHLSE GLDNRGSGIRHISVFVCREVN.	Up

**a40**	gi|91077292	NLU004080.1	1.406048059	1.137627006	Aplicing factor 3A subunit 3 *[Tribolium castaneum]*	ELVSEQRTATKENVQRKQARTEGERDDSDAEISASDSDEDDEDAVPYNPKNLPLGWDGKPIPYWLYKLHGLNISYNCEICGNFIYKGPKAFQRHFAEWRHAHGMRCLGIPNTAHFANVTQIEDALSLWEKLKSQKQEERWQPEQEEEFEDSLGNARHTNNWAVLVDTSRFWFNYRHVANVLSIYRSVKRLGIPDSQIILMVADDMAFYPPNPRPACNPRNPRPATVFNNANQHINVYGDDVEVDYRGYEVTVENFVRLLTGRLPADTPRSKQLLTDEGSNVLVYLTGHGGDGFLKFQDSEEVTSQELADALEQMWQKRRYHEIFFMIDTCQAASMYEKFYSPNILAVASSLVGEDSLSHHVDPAIGVYIIDRYTYYALAFLETVQPDSMKSMAEFLAVCPKRVCISTVGVRQDLFARNPHSVPITDFFGALRPVQLTFSSLVLPENQCFDCEKINITSSVK.	Up
**a41**	gi|328701792	NLU019400.1	1.30617094	1.224616051	Protein kinase 3-like *[Acyrthosiphon pisum]*	MCVFVAQKSVLLAELIGTECYYAVKCLKKDVVLEDDDVECTLIERKVLALGTNHPYLCHLFCTFQTESHLFFVMEYLNGGDLMFHIQQSGRFDEGRARFYASEIVSGLMFLHKKGIVYRDLKLDNILLDFDGHVRIADFGMCKLQIYLDRTADTFCGTPDYMAPEIIKGLAYNQCVDWWSFGILLYEMLVGQSPFSGCDEDDLFWSICNEQPHYPRFLSKESKLILSQLLEKDSSRRLGSSELSGREVTSHVFFRDWDWPRLERRQLDPPFKPRVRHPLDVQYFDKAFTNERPRLTPIDKSILQSMDQTQFQGFSYTNPNVTK.	Up
**a42**	gi|307184734	NLU015557.1	1.355188966	1.213389039	Nuclear protein NP60-like protein *[Camponotus floridanus]*	MYVIRQVTPPLDLENVSETLKEKNIKPSALRFGFLGLGIMGSGIVKNLLNSGHSVIVWNRTPDKMIWTELLALSFVNREIIAGLINAVTCLIKADINYSTTSKFCRPIVRFCNKKRYLRWEILSFIELQSVALHTDE.	Up
**a43**	gi|642930478	NLU022689.1	1.393157005	2.703958035	Aodium/potassium-transporting ATPase subunit alpha isoform X3 [Tribolium castaneum]	HWLDAVIFLIGIIVANVPEGLLATVTVCLTLTAKRMASKNCLVKNLEAVETLGSTSTICSDKTGTLTQNRMTVAHMWFDNQIIEADTTEDQSGVQYDRTSPGFKALARIATLCNRAEFKGGQEGVPILKKEVNGDASEAALLKCMELALGDVMSIRRRNRKVCEVPFNSTNKYQVSIHETEDANDPRHLMVMKGAPERILDRCSTIFIGGKEKLLDEEMKEAFNNAYLELGGLGERVLGFCDFLLPSDKFPLGFKFDCDDPNFPISGLRFVGLMSMIDPPRAAVPDAVAKCRSAGIKVIMVTGDHPITAKAIAKSVGIISE	Up
**a44**	gi|746841002	NLU021944.1	1.180320978	1.419057012	Polypyrimidine tract-binding protein 1 isoform X1 *[Acromyrmex echinatior]*	MLDKAKLDKTNSIKPSKVIHIRNIPNEVTEAEIIHLGIPFGRVTNVLVLKGKNQREKEREKKNKKKIEDKQEEYEDKKKRKKKKQKRKKEKEQKRKKKMDKHLIQECNIIDFLKQE.	Up
**a45**	gi|111145227	NLU021961.1	1.406048059	1.127197981	Enolase *[Drosophila elanogaster]*	HIADLAGNKEIILPVPAFNVINGGSHAGNKLAMQEFMILPTGATSFTEAMKMGSEVYHHLKNVIKAKFGLDATAVGDEGGFAPNIQSNKEALNLISDAIAKAGYTGKIEIGMDVAASEFYKDGQYDLDFKNEKSDKSQWLPADKLANLYQEFIKDFPIVSIEDPFDQDHWEAWSNLTGCTAIQIVGDDLTVTNPKRIATAVEKKACNCLLLKVNQIGTVTESIAAHLLAKKNGWGTMVSHRSGETEDSFIGDLVVGLSTGQIKTGAPCRSERLAKYNQILRIEEEIGAGVKFAGKSFRKPQ	Up
**a46**	gi|328720754	NLU018304.1	1.819700956	32.80952835	Probable low-specificity L-threonine aldolase 2	ALCDKEAGLYVSSGTMGNLVAIMAHTAHVRGADIIVGSKCHIVRYEQGGAAQIGGVTLNVVPNNPDGTFDIDEMLTQIHEGEHDVHYPVTALICVENTHNACGGRILPLSWLSSIVEIGKEKNIPLHMDGARLFNAVVAQGVSAAEITRGFSSVSICLSKGLGAPVGSVLVGSQDFIKKARRIRKVL	Up

					[Acyrthosiphon pisum]	GGGMRQVGVIAAAGLYAVENNIKRLAEDHYNTLTLANAIAELKSDLVKVNLEVVETNILFIYFDNSQLTPEEFCSRMVKVTEEERNALGDND VCAVQMFSMDSFSARIVLHSNLTARDVKLAMKKFQFVIKEFMSRTL.	
**a47**	gi|383865623	NLU008406.1	1.355188966	1.270573974	28 kDa heat- and acid-stable phosphoprotein-like *[Megachile rotundata]*	KAKGVENLIEVENPNRVQKKTKKSSALNEAILKEDVKPNLSRKEREELDRQRATQHYQKLHAEGKTDEARADLARLAIIKQQREEAAKQRD LEKQSE.	Up
**a48**	gi|641673056	NLU008141.1	39.4457283	1.599557996	Laminin subunit beta-1 *[Acyrthosiphon pisum]*	MLIERSYDFGRTWKVYRYFAQNCDESFPGVSKDSQKTLTDVVCDSRYSSVAPSSDGEVILRVLPPNLHHLYDDPYSPDVQNLLKMTNLRINFT KLHTLGDDLLDNREEIQQKYYYAVREMVVRGSCSCYGHASKCLPQDGSEDLNPDMVHGKCECTHNTQGLNCEQCKDFYNDLPWKPAIEKQ TNACKECNCNGHSRRCHFDQNVYEMTGYVSGGVCDGCMHNTVGRNCEQCMPQFFYDPIRNYNESDACQRKFSFKPVDSHYDSF.	Up
**a49**	gi|17137546	NLU006367.1	1.202263951	1.786488056	Flotillin,isoform A *[Drosophila elanogaster]*	GRAFVWPVGQQVQRISLNTMTLQVESPCVYTSQGVPISVTGIAQVKVQGQNEDMLLTACEQFLGKSEAEINHIALVTLEGHQRAIMGSMTVE EIYKDRKKFSKQVFEVASSDLANMGITVVSYTIKDLRDEEGYLRSLGMARTAEVKRDARIGEAEARAEAHIKEAIAEEQRMAARFLNDTDIA KAQRDFELKKAAYDVEVQTKKAEAEMAYELQAAKTKQRIKEEQMQVKVIERTQEIAVQEQEIMRRERELEATIRRPAEAEKFRMEKLAEAN KQRVVMEAEAEAESIRIRGEAEAFAIAAKAKAEAEQMAMKAEAYREYREAAMVEMLLDTLPKVAAEVAAPLSQAKKITMVSSGTGDIGAA KLTGEV	Up
**b1**	gi|292606981	NLU016425.1	0.758577585	0.613762021	Heat shock protein 90 *[Nilaparvata lugens]*	DSEDLPLNISREMLQQNKILKVIRKNLVKKCLELFEELAEDKDNYKKFYEQFSKNLKLGIHEDSQNRKKLSDLLRYHTSASGDDNCSLKDYV GRMKENQKHIYYITGESKDQVANSSFVELVKKRGFEVVYMTEPIDEYVVQQMKEYDGKQLVSVTKEGLELPEDEAEKKKREDDKAKFENL CKVMKDILDKKVEKVVVSNRLVESPCCIVTSQYGWTANMERIMKAQALRDTSTMGYMAAKKHLEINPDHSIIDTLRTKADEDKNDKAVKD LVMLLFETALLSSGFALEDPGVHAARIHRMIKLGLCIEEDDPVPHDDEKVDAEMPPLEGEASEDASRMEEVD.	Down
**b2**	gi|209571429	NLU010815.1	0.809095919	0.48305881	Lipophorin precursor *[Nilaparvata lugens]*	APHIAYLGIGSFAGRFCNLHDCQNVPEFKELLSKLAAPVFAGCKVDSIENENKLIASLKGLHNVHHLTDEVAGKLAACAQDKSVKTRIRVAAL EAFQSDASKSKIREASIAILKDLSEDSEIRIKAYLSLVECPCNKVADVIKNVLDNEKSNQVGSFIVSHLRNVRASTNPDKAHAKHFLGAIHTGN KFPIDVRRFSTNHELSYSLDAVNVGTAAEGNVIFSEQSFVPRSVNLNLTTQVFGHAFNLFEVNARSENIDYLLEKYFGPKGYFPTHNPHDIFDT VAKTSKSLADKIKERYENTIRPKRSIQEQTSAFAKNVVVGGVDSHDKNLDIDLSLKMFGAEVGWYTYTGSSKKFDMSFIDNIFNKVDSSLDK AKNFDRLNGEP	Down
**b3**	gi|642938341	NLU004755.1	0.452897608	0.613762021	Leucine-rich	MFKRPGAKRHASAGRVKSAIKRAHARGNGGQAFSGGHRPQGRGGCQGDEEAVLEEAEDEEVENNQIEELPKEIGQLQHLKTLKVKMNRLK	Down

					repeat-containing protein 40-like *[Tribolium castaneum]*	SVPEELFKLKEIVHLDLSENLICEIHEGLGDLVTLQKLDLSRNKLSAVPLSLGYLTSLVTLNISHNHLASVPEEIQNLRGLTDLDISHNNISELPPMTHMSHLTIIEASCNQIADVPAFNNCRKLREVRFGQNKIEKIEVGNFEEVPVSVLILSSNQILEVPEDIARLTTLTILDLSKNSITNIPPIMTRLRKLTHLKLEGNPIKNIRQDILKTGTLRLLDHLSKKLKDEIASQGNPNNSMHFYDACYSEESDVMLDKQEMRSGNLILCNSNMAKIPEEIISQAQDAKILSVDLSKNNFTIFPDNITALQATLSELDLKFNFLEELPSCIGQLSQLQFLNLSHNKLTTLPNELNQCLRMREILLESNRLSEIPDVIHDLTHLERLDVAGNSINEINVDRLGNLKRLYYLNVSCNNIQKIPCELGKLEHIKRCSLQSASAAWSWVRILPVKKVVVPRDLKDWELNFIKGFLKSFFPNFDSFKINKKDKSN	
**b4**	gi|157115817	NLU028181.1	0.428548515	0.390840888	Alanyl-tRNA synthetase *[Aedes aegypti]*	LVTVVGESAGAASAHFHILSPQSQGLFQRAILLSGTADCPWAVSTAHQNGNLTAKMASLVNCSADTSATELLECLRKVEGSEFLIHNEKFQTVWKGNSVPIVIFRPTIESHSGNAFITQESYKGQSKKPMMIGATSDEGALVLAILKRDKTRSLESALSEFDKRFTEIMPVEGDFLDDPDHKERAEKIKTEYFGNSTISNETLPQLTKDMSEALKLISCVLKTLKPDLSQPGVCHADDLFYLFPMKPFLGLRVGSETEKDKEISAKFVDLITNFVIEGCLQVSEFLPGNQVEGSEDCLYLNVYTPSRNGVGYPVMVFIHGGGFVGGAATSDLYGPDKLLLTKDIILVTIHYRLGFLGFASFDDRDFAGNYGLKDQSLALKWVKENIAKFGGDGDKVTVVGESAGAASAHFHILSPQSQGTPHQINSHLILVVL.	Down
**b5**	gi|153792659	NLU019724.1	0.478630096	0.642687678	Actin-depolymerizing factor *[Bombyx mori]*	MASGVTVSDACKTTYEEIKKDKKHRYVVFYIRDEKQIDVETVGERNAEYEQFLEDLQKGGTGECRYGLFDFEYTHQCQGTSEASKKQKLFLMSWCPDTAKVKKKMLYSSSFDALKKSLVGVQKYIQATDLSEASQEAVEEKLRATDRQ	Down
**b6**	gi|170056920	NLU001990.1	0.895364821	0.772680581	Disulfide isomerase *[Culex quinquefasciatus]*	QAFKDQGIEEDSKIVLFKQFDEGKAIFDGELDEANLKKFVIAESLPLIVDFNQDTATKIFGGEFQSHLLLFLSQSSGHYDTLLEGAKSIVKEYRDKILFVSINADEEEHGRILDFFGLKKEEIPSMRIIKLADDMVKFKPEKQDFSAENIKSFVSDFIEGKLKQHLLSQDLPEDWDKKAVKVLVASNFADIAYDKSKDVLVEFYAPWCGHCKQLEPIYDQLGESLKEKDDIVVAKMDATINELEDTKILSFPTIKLYKKGDNKVVQYNGERTLEGLSKFIESGGEYGKAPAEAEEEDEDDDLPRKDEL.	Down
**b7**	gi|91087369	NLU017666.1	0.717794299	0.724435985	Translation elongation factor 2 *[Tribolium castaneum]*	KELQLESEDLYQTFQRIVENVNVIIATYSDDSGPMGEVRVDPSKGSVGFGSGLHGWAFTLKQFAEMYAEKFKIDVVKLMNRLWGENFFNPKTKKWAKQKEEDNKRSFNMYVLDPIYKVFDCIMNYKKDEAAVLLQKLGIELKPEDKEKDGKQLLKVVMRTWLPAGEALLQMIAIHLPSPVVAQKYRMEMLYEGPHDDEAAIGVKNCDPNAPLMMYISKMVPTSDKGRFYAFGRVFSGKVATGMKARIMGPNFVPGKKEDLYEKAIQRTILMMGRYVEAIEDVPSGNICGLVGVDQFLVKTGTITTFKDAHNMRVMKFSVSPVVRVAVEPKNPADLPKLVEGLKRLAKSDPMVQCIIEESGEHIIAGAGELHLEICLKDLE	Down
**b8**	gi|170062187	NLU016473.1	0.658967585	0.646767521	pyruvate dehydrogenase *[Culex quinquefasciatus]*	AQQLTVRDALNAALDEEMERDERVFILGEEVAQYDGAYKVSRGLWKKYGDKRVIDTPITEMGFAGIAVGAAMAGLRPVCEFMTFNFSMQAIDQVINSAAKTFYMSAGTVNVPIVFRGPNGAASGVGAQHSQCFGAWYSHCPGLKVVAPYDSEDAKGLLKAAIRDPDPVVVLENEMVYGQGFPVSDQVLDKEFVLPIGKAKIMRPGKHVTLVAYAKAVETAMLAANELAGKGIECEVINLRSL	Down

**b9**	gi|94468780	NLU023845.1	0.724435985	0.316227794	Translation elongation factor EF-1 alpha/Tu *[Aedes aegypti]*	MGKEKVHINIVVIGHVDSGKSTTTGHLIYKCGGIDKRTIEKFEKEAQEMGKGSFKYAWVLDKLKAERERGITIDIALWKFETAKYYVTIIDAPG<br>HRDFIKNMITGTSQADCAVLIVAAGTGEFEAGISKNGQTREHALLAFTLGVKQLIVGVNKMDSTEPPYSENRFEEIKKEVSSYIKKIGYNPATV<br>AFVPISGWHGDNMLETSDKMPWFKGWNIERKEGKAEGKCLIEALDAILPPSRPTEKPLRLPLQDVYKIGGIGTVPVGRVETGVLKTGMVVTF<br>APANLTTEVKSVEMHHEALVEAVPGDNVGFNVKNVSVKELRRGYVAGDSKSNPPKAAADFTAQVIVLNHPGQIAAGYTPVLDCHTAHIACK<br>FAEIKEKCDRRSGKTTEENPKSIKSGDAAIITLVPSKPMCVESFQEFPPLGRFAVRDMRQTVAVGVIKSVTQKDLSSGKVTKAAEKAQKKK.	Down
**b10**	gi|751804680	NLU003167.1	0.654636085	0.816582382	Probable ATP-dependent RNA helicase DDX43 [Bactrocera dorsalis]	MLAKTYMKDPVSVHVGSLDLTATHTVEQVIEIMTEEEKEEALMSLVANMQPDDKMIVFVGKKVRADALSTDMALRGVDCDCIHGDREQSD<br>REQALAALKSGSVRILIATDVASRGIDIPDVTHVVNLDFPRCIEEYVHRVGRTGRAGREGQAISFVTRQDWGQARELITILEEAGQVVPEELEQ<br>MAERFAAMKQQRSEGGGGGGFRGRRGGGGGYGGGGGGGYGGGGGGGYGGGGGGGYGGGRGRGGGGRGGFRGSRRGGDSW.	Down
**b11**	gi|540361100	NLU026472.1	0.801678121	0.744732022	Arginine kinase *[Nephotettix cincticeps]*	MVDAAVLEKLETGFKKLQESDSKSLLKKYLTQEVFDSLKTKKTPTFGSTLLDVIQSGLENHDSGVGIYAPDAEAYGVFADLFDPIIEDYHGGF<br>KKTDKHPPRDFGDVDSFANLDPAGEYIISTRVRCGRSMQGYPFNPCLTEAQYKEMEEKVSSTLSGLSGELKGDFYPLTGMTKEVQQKLIDDH<br>FLFKEGDRFLQAANACRFWPTGRGIYHNDNKTFLVWCNEEDHLRIISMQMGGDLGQVYRRLVSAVNDIEKRIPFSHDDRLGFLTFCPTNLGT<br>TIRASVHIKVPKLAANKAKLEEVAGKFNLQVRGTRGEHTEAEGGVYDISNKRRMGLTEYQAVKEMNDGIAELIKIEREL.	Down
**b12**	gi|641675408	NLU001600.1	0.839460015	0.711213529	Fatty acid synthase *[Acyrthosiphon pisum]*	VLEKYQFVPHVNNEKSDINDIVRICVHLVLENVPSQKVKTAEVLEPSVESNTPVLTPIINKTLGDLPLIQADNSILSDVNNPLLADLSSEILIEDK<br>KLTPDQTLLLLVISNVLSAHRHQALKAALASVKESAFILAREDCKTTPVIPAELKLCAVFQTENEKLYLIRKVQKVESAPVIINIPEDNFSWLPIL<br>KAAVKAEDVPSDQRIIVYAQNEPFNGIIGLVNCLRREGSGEKIRSFLILDKNAPPFDVSNPFYKEELSKDLAVNVYKDGKWGSYRHLLLKENSL<br>VEAHHSY	Down
**b13**	gi|225031000	NLU028132.1	0.765596628	0.534564376	Myosin 2 light chain *[Nilaparvata lugens]*	MADVSSDDVERATFAFNIYDTEGGGNLDAFFLGDLLRALGLNPTQGLIEKLGGTKKKGEKTFTFEEFLPIFSQARKDKDNGVYEDFIECLKLY<br>DKAENGLMVGAELSHILLSLGER	Down
**b14**	gi|91087635	NLU019210.1	0.496592313	0.608134985	proteasome zeta subunit *[Tribolium castaneum]*	MFLTRSEYDRGVNTFSPEGRLFQVEYAIEAIKLGSTAIGICTSEGVVLAVEKRITSPLMEPTTIEKIVEVDKHIGCAVSGLMADSRTMIDRARVEC<br>QNHWFVYNENMSVESCAQAVSNLAIQFGDSDDDGAAMSRPFGVAILFAGIDEKGPQLYHMDPSGTFVQFDAKAIGSGSEGAQQSLQEVYHR<br>SMTLSEATTAALTILKQVMEEKLNSSN	
**b15**	gi|156547575	NLU015620.3	0.580764413	0.698232412	Putative ATP-dependent RNA helicase me31b *[Nasonia vitripennis]*	LAKHMNVRVMVTTGGTNLRDDILRIYQKVHVIIATPGRILDLMDKNVANMDNCKILVLDEADKLLSQDFKGMLDHVISRLPHERQILLYSATF<br>PLTVKQFMEKHLKEPYEINLMEELTLKGVTQYYAFVQERQKVHCLNTLFSKLQINQSIIFCNSTQRVELLAKKITELGYCCYYIHAKMAQAHR<br>NRVFHDFRAGLCRNLVCSDLFTRGIDVQAVNVVINFDFPKMAETYLHRIGRSGRFGHLGIALNLITYEDRFALHRIEQELGTEIKPIPKVIDPDL<br>YVAKLDESIEDANVSK.	Down
**b16**	gi|641676253	NLU007745.1	0.648634374	0.602559626	Asparagine synthetase	QTFAIGMGESPDILAARQVAAHLGTEHHEISFSAEDVASVLDEVIFHLETADITTIRASVGMYLLSRYIKEKTDSTVIFSGEGADEVCQGYIYFR	Down

					[Acyrthosiphon pisum]	DAPDAAAGDRESRRLLSDIYMYDGLRADRTTAAHSLELRVPFLDQEFTHYYLNLPPEVRQPRDGVEKFLLRSAFDKMNLIPDSILWRHKEAF<br>SDGVASIKKSLFQVIQEFVDPKVPNDALNSAEKLYPHCTPKTKESFYYRKVFEKFYHDQAGWLVPYYWMPKWTNVSDPSARFIKHYAAKDD<br>GS.	
**b17**	gi|91081061	NLU010039.1	0.801678121	0.870963573	40S ribosomal protein S2<br>*[Tribolium castaneum]*	MADAAPAGRGGFRGGFGGRGGAGAGGRGGMRGRGRGRGRGRGRGKEAEKEWMPVTKLGRLVRDGKIHSLEDIYLFSLPIKEYEIIDQFIGS<br>SLKDEVLKIMPVQKQTRAGQRTRFKAFVAIGDLNGHIGLGVKCSKEVATAIRGAIILAKLSVVPVRRGYWGNKIGKPHTVPCKVTGKCGSVQ<br>VRLIPAPRGTGIVGAPVPKKLLQMAGIEDCYTSARGSTGTLGNFAKATYAAIAKTYAYLTPDLWKESVLKKTPYSEFADYLFKNHRPVLAVTQ<br>DN.	Down
**b18**	gi|157105307	NLU009392.1	0.648634374	0.666806817	acyl-coa dehydrogenase<br>[Aedes aegypti]	AALSQLIKTAVRPACRALSSKAAPAVEASSMGPSFAMSDEQKEIVDMTKKFVREEIIPVAGQYDKTGEYPWPIIRKAWELGLMNNHIPADIGGT<br>DMSIMTSCMIAEELAYGCTGIMTALEASGLGQTPVIIAGNPEQKKKYLGRLLEEPLVAAYCVTEPGAGSDVNGVKTRAEKKGDEYILNGQKM<br>WITNGGVANWYFVLARTNPDPKCPASKAFTGFIVERDTPGLTPGRKEQNMGQRASDTRGITFEDVRVPKENVLIGEGSGFKIAMGTFDKTRPP<br>VAAGAVGLAQRALDEAMKYSLERKTFGVPIAAHQAVSFMLADMAIGVETARLITMKSAWEVDQGRRNSYYASIAKCYAADMANKVASDAV<br>QIFGGNGFNTEYPVEKL	Down
**b19**	gi|723001888	NLU019204.1	0.824138105	0.73790431	Vitellogenin<br>*[Nilaparvata lugens]*	ISGADAMVVDVSARFQDSHGQSNAQYVATVAMANSDASPNARMLFFASMNPANSDSKAQVCAAVASNFPNVPLMNFHDALKANPTSRISA<br>DIAFGAQCNAGGHIHADAKLSQTQEFQEYTKSRPMAKKCFQLMEKGQALEYACQNATKVANMLNNYDVSVKYDRVPNALKNITYSIYSAL<br>AQAAYPYHNKNMFSQNSNPAGRIEANVRFNNNLHYFNASINTPFFSANVKNVEVHHALRPLVIFHPSLNSLELMSYNENYDYPTCSVSKNSIS<br>TFDNKTYSADLEGWHVMFASTPKNYNDNSGRYSASNSQSNSFYKYKKVVVLAKNAGSQRKAVKMLLGENVIDINPSGSESSDNSPNANVQ<br>VNGNKVQIANNRMASFDDFDGETLVEISVTDNGEVQVQSSSHGIAVYHDGANFIIDADSYHRGEVRGLCGTYSGDKYTDFTTPNKCIMREAR<br>LFAATYALIFADVITSNDYDRSSSSSSSSSNRNNNKNNRNNNNSSELRTNPTKLVQDIKNNGDHVCFSIHPVPKCQSGFSPAESSEKEVQYLCIS<br>KGKNAEYWVGQISNGGFVNLGQKQPNATFKKSERPKELC	Down
**b20**	gi|642928254	NLU001358.1	0.660693526	0.510505021	Bifunctional glutamate/proline--tRNA ligase<br>*[Tribolium castaneum]*	CPIVDSIEGVTHCLRTMEYHDRDEQFYWFIDALSLRKPHIWEYSRLNMTNTVLSKRKLTWFVENAFVDGWDDPRFPTVRGVLRHGMTVEGL<br>KQFIIAQGSSRSVVVMEWDKIWAFNKKEEQLVVAKHPKNPAVGTKTVCVGPRILIDYADAECLKEGENTTFINWGNLRIKKINRKDNRIVSVD<br>AEPNLDDKDYKKTLKITWLADSSKAPLIPALITYFDHIISKPVLGKDEDFKTYIGHNTKTEVKMLGDPELANVKKSEIIQLQRKGFFICDSPYKP<br>ASIHSGRESPIC	Down
**b21**	gi|226442059	NLU005951.1	0.758577585	0.870963573	Heat shock protein 60<br>*[Pteromalus puparum]*	ETTSEYEREKLQERLARLASGVAVLKVGGSSEVEVNEKKDRVTDALNATRAAVEEGIVPGGGTALLRCAAILKQVITANADQATGIDIVQKAL<br>RMPCMTIAQDAGGDASVIVSKVAEGTGDMGYDALNNEYVNMIEKGIIDPTKVVRTALTDAAGVASLLTTAEAVVAELPKEEPAMPAMGGMG<br>GMGGMGGMGMGM.	Down

**b22**	gi|642913258	NLU020051.1	0.636795521	0.724435985	RNA-binding protein squid isoform X3 *[Tribolium castaneum]*	MADSTLEYDMSNGEFQQGGEDYTQDDQMNGSGENGGANEGGDSGSAEAPGKDDERKLFVGGLSWETSDKELRDHFGTFGEIESVNVKTD PNTGRSRGFAFIVFKSADAIDKVVNAGDHVINNKKIDPKKAKARQGKIFVGGLSAELSDDDIKSYFAQYGTIVQVDMPYDKSKNQRKGFCFIT FESEQIVTEVLKTPKASIKGKEVDVRKATPKPDGMGNTRGGRGAGRGTRGRGRGGAGGYGTGYDYYGSGGYGGGYGGGYGTYDYSGYG GYGSYDGYGAYGNNYETGYGTGGGGRGGRGKGTGGGYGGGGKQRGGGRGQRHTPY.	Down
**b23**	gi|641676959	NLU016669.1	0.879022479	0.765596628	Aldehyde dehydrogenase, *[Acyrthosiphon pisum]*	VDRFIGPTVLVDVKPTDPVMKEEIFGPILPIVNVENAYEAIGFINSREIPLTLYVFTNKSADMQLFLKHTSSGSVCCNDTAVHLAVETLPFGGVG HSGMGAYHGKYTFDTFTHKKSCLVKDYNPLLESLASSRYPPYSDKKMNFIAMLMRKRRSLGLQYIPYAMTFAVGVLTAVAFMKFYKVVPYS RRP.	Down
**b24**	gi|91083095	NLU014434.1	0.679203629	0.586138189	40S ribosomal protein S4 *[Tribolium castaneum]*	MLDKLGGVYAPRPSTGPHKLRESLPLVIFLRNRLKYALTNCEVKKIVMQRLIKVDGKVRTDPNYPAGFMDVVQIEKTNEFFRLIYDVKGRFTI HRITAEEAKYKLCKVKRVQTGPKGIPFLTTHDGRTIRYPDPLVKVNDTIQLDIATSKIMDFIRFDSGNLCMITGGRNLGRVGTVVNRERHPGSF DIVHIKDVLGHTFATRLNNVFIIGKGSKAYVSLPKGKGVKLSIAEERDKRLAAKASSG.	Down
**b25**	gi|157134829	NLU008827.4	0.787045777	0.01318257	AMP dependent coa ligase *[Aedes aegypti]*	GAAPAGASDIERCFQKMPKSFIFMQAVTKKQLEHMRFLASGAAPIGASDVTRTLHKMPESCIFMQAYGLTETSPVCLLPRNNNRNLSTVGSPT SITKAKIIDTSTGKILGPNEHGELCIYGPQVMQGYLNNQKATDETIINGWLHTGDIGYYDDEGLFYIVDRLKELIKVKGFQVAPAELEELLRTHP KVNDVAVIGVPDARSGEVPLAYVIRKDDVTEDELKGYISDRVAPFKQLAGIVFTDSIPKSPSGKILRRFLKDAYLKEHKK.	Down
**b26**	gi|91079082	NLU012202.1	0.831763685	0.666806817	Translocon-associated protein subunit alpha *[Tribolium castaneum]*	MRRLCCQDSGIANINVFFFQFEADYFLPPRRQFGRLMCIAYASPCPRLFALADEVEDEEDDELVDVGEETDGEETAMTDEGTEEEEDSSGSKS GGSPNADTTILFIQPIAQPGSTLDLPAGKIVEFLVGFLNKGDEDLILETLDASFRYPMDFKFYIQNFTTLVYERPVKPGHEATLSYSFIPAEAFASR PFGLSVNLRYRDDSGNQFYEGVYNETVNIVEIEEGLDGETFFLYVFLGACGVLLLVLGQQFLVSVGKKRVGGGTTTKRPQVETGTTNPNDVD YDWLPRETLQSLNKSPRARPVAKQQSPKQRKAKRTAGSDD.	Down
**b27**	gi|149689182	NLU021085.1	0.824138105	0.648634374	Ribosomal protein P2 *[Triatoma infestans]*	MVKNIQLAFFEDTVENPSSGDIEKILSSVGIEADSEKLNFVVGQLKGKSVDELIEQGKEKLASMPAGGGAAVAVAAGGAPAAAAAAPEKKEE KKAEKEESDQSDDDMGFGLFD.	Down
**b28**	gi|240849631	NLU026824.1	0.666806817	0.801678121	ATP synthase subunit b, mitochondrial-like *[Acyrthosiphon pisum]*	MLADLVDCLSAKQVRPALIGAVRCSGTEAATSQSQQLMSKVAVMAPAASRQLSATATGDRPPWEGPERDLVNFPRPVMPEEPGKVRYLFVPE EWFEFFYKKTGVTGPYVLAFGIFNYVMSKEVWIIEHEYYYVYALAAIFYFGDKKFGKQIGDYLDKEIEADTHFFEKDRLDKIANFNKSIEEEK TLQWQKEADKLIIEAKRENVALQQEAIFRERAMFAYQEIVYYATSTESLCLRYEDSFPGEACRAREYVIRIP.	Down
**b29**	gi|478896864	NLU012724.1	0.539510608	0.501187205	Glutamine synthetase 2 *[Nilaparvata lugens]*	MSGILENSPNYKLNKTVLDKFLSLPQPENKVQATYIWIDGTGEGIRAKTRTLDFVPKSVSETNHRAACLEAMTKAAAQKPWFGIEQEYTLLD MEGRPFGWPRNGFPGPQGPYYCGVGANKVYARDVVEAHYRACLYAGINISGTNAEVMPSQWEFQVGPCEGIEIGDQLWVARYLLHRVAEEF GVVVTLDPKPMEGDWNGAGAHCNFSTKTMREENGIIEIEKAIDKLSKQHIRHIKAYDPKEGKDNERRLTGKHETSTIHDFSAGVANRTASIRIP RGCAEEKKGYLEDRRPASNCDPYSVANVIVRTVCLNE.	Down

**b30**	gi|189241162	NLU020749.1	0.895364821	0.847227395	26S proteasome non-ATPase regulatory subunit 11 *[Tribolium castaneum]*	MSKTERPRKEEADDECAIRTKIKMAGAMLFERAVSTPSRDDLHKNLVIQGEIEENEEEDIKQREAGILQLGEQYKNEGKAKELAELIKATRPFL SLISKAKAAKLVRSLVDFFLDLEAGIGIEVQLCKECIEWAKEERRTFLRQSLEARLVALYFDTGMFTEALTLGSNLLKELKKLDDKNLLVEVLL LESKTYHALSNLSKARAALTSARTTANAVYCPPKMQAALDLQSGILHAAEEQDFKIAYSYFCEAFEGYDSIESNKALTALKYMLLTKIMLNTP EDVQQIISGKTALKYGGKDIEAMKSVAQASHKRSLADFQKVLKNYKKELEEDPIVRAHLGSLYDNMLEQNLCRIIEPYSRVQVEFISQAIKLPT LQVEKKLSQMILDKKFHGILDQGEGVLIVFEEKAMDKTYEMALETITSMGKVVDTLYQKAKKLS.	Down
**b31**	gi|91086093	NLU019302.1	0.544502676	0.73790431	Tubulin beta-1 chain *[Tribolium castaneum]*	NATLSVHQLVENTDETFCIDNEALYDICFRTLKLTSPTYGDLNHLVSVTMSGVTTCLRFPGQLNADLRKLAVNMVPFPRLHFFMPGFAPLTAR GSQQYRALSVSELTQQMFDAKNMMTACDPRHGRYLTVAAIFRGMMSMKEVDEQMLNVQNKNSGYFVEWIPNNVKVAVCDIAPRGLTMSA TFIGNTTAIQEIFKRISEQFTAMFRRKAFLHWYTGEGMDEMEFTEAESNMNDLVSEYQQYQEASAEDDVEFDEEEVIPEEQEQEPEA.	Down
**b32**	gi|91093363	NLU018217.1	0.765596628	0.88715601	Proliferation-associated protein 2G4 *[Tribolium castaneum]*	RETNQGADGQAYSITNAVQSVCDDFKCKPVEGMLSHQLKQFKIDGEKTIIQNPTEAQKKEHEKHEFAVHEVYAMDVLVSTGEGVGKEADTK VSVYKKTDETYQLKLKASRMFYSEVCHKYGTMPFNLRNFEEETKAKMGVGECVKYKLIEPFQVLYEKPSEFVVHFKFTVLLMPNKPHKITG LDFTPELYQSEHSVSNQEMKTLLCCPTTNPKSAKKKKKKTNKSGDDDKNEEVNDVANRINDVKI.	Down
**b33**	gi|723001902	NLU007082.1	0.539510608	0.724435985	EF-hand motif protein *[Nilaparvata lugens]*	MSLSRVIRTRASRNNLILGMETDIDGLKEGLINNAVIDIAIDLHATLVDICDKVSNEAVIDLIPLITSLLNNYDAALKVNSDLKNMLNDVNFEKE ALIERLRIERENRTMIFEDSLSNEKQAESEICALKLRVKDLEEAVHSFKLEIASKEEIINPVKMLRLQLVVVGVALIACCCANPPTPTPRSFPKFKP EDTTDFKTDVRIQRNTKLKTETEKKTNHSAMFKVKKYSPLIHSVMLFFDILSFKSKVAARAAAGTDGSVNRAQFKTACIGLYAQRTSPQLTPT DQEIEALFKLYDRDHNDKIDMKEGIMGVMQHQEFFYYDMNLDGKLDENELMYMYDTRDQNMQYSGSADFVGKFFYLYYPI.	Down
**b34**	gi|91085777	NLU023681.1	0.801678121	0.597035289	60S ribosomal protein L23 *[Tribolium castaneum]*	MSKRGRGGSAGAKFRISLGLPVGAVINCADNTGAKNLYVIAVQGVKGRLNRLPAAGSGDMIVATVKKGKPELRKKVMPAVVIRQRKPFRRK DGVFIYFEDNAGVIVNNKGEMKGSAITGPVAKECADLWPRIASNASSIA.	Down
**b35**	gi|307189141	NLU008388.1	0.862978518	0.794328213	Serine/threonine-protein phosphatase 2A 65 kDa regulatory subunit A alpha isoform *[Camponotus floridanus]*	KSDLVPAFQSLLKDSEAEVRAAAAHKVRDFCHNLDAYQKEEIIMKNILPFVKELVTDPNQHVKSALASVIMGLSPILGKHNTIEHLLPLFLLQL KDECPEVRLNIISNLDCVNEVIGIQQLSQSLLPAIVELAEDTKWRVRLAIIEYMPLLAGQLGVDFFDEKLNSLCMSWLLDHVYAIREAATLNLK KLVEKFGADWACNTVIPKIQFMARDHNYLHRMTCLFSINVLAGVCGPEITEKLMLPTVIEMAFDSVANVRFNVAKTLQKISPVLDPNVIQAQV KPVLDQLNTDSDVDVKYFASEAIAGIAGITNHSANTAK.	Down
**b36**	gi|91088575	NLU021401.1	0.544502676	0.505824685	Ubiquitin conjugation factor E4 B *[Tribolium castaneum]*	LMNDTTFLLDESLESLKRIHEVQELMSDTAAWKALSTEQQQVRNRQLNADERQCRSYLTLARETVDMFHYLTVDIKEPFLRPELVGRLSAML NFNLQQLCGPKCKNLKVQQPEKYGWEPRRLLGQVGDIYLHLDCEQFAAAIAADERSFRKELFDDAAVRMERALIKTSTDIEKFRNLIQQATEI AIQNIKREVDYNDAPDEFRDPLMDTLMDDPVQLPSGKVMDRSVIVRHLLNSSTDPFSRQPLSEDMLKPDKSGKTPVAAVAVDGYTAAANGNI	Down

						AARPFCRPGCKLPFQSIDYCSSGGDGEITAPAEPAQCVTGVRRSLKEGWDPGLTIKDGIIAGGGGGSCRSRPGRQSP.	
**b37**	gi|193636528	NLU017187.1	0.847227395	0.88715601	Protein takeout-like *[Acyrthosiphon pisum]*	MLTDINSIHSVAQYIKPCKIDATLDDCALKNGRIAIPKLLNGDSKYKIPKLDPFQIKSLTVDHGSKQIGLKLEMIDSLLTGLKHIKFTASRMDMKKRHIEWDVLVPKLELFGKYKADGKILVLPIVGNGNGNITLVNLNATYSFDFNKEKRNGKDYIKVMSSKIKFDCSRLYFKLDNLFNGDKLLGDNMNQFLDENWREVLNELGDPIAAPIAEIFTVILKGVVNEIPFDEVFLK.	Down
**b38**	gi|91079450	NLU013993.1	0.698232412	0.724435985	Venom serine carboxypeptidase *[Tribolium castaneum]*	MFPEIQNAPFYITGESYAGKYIPALGYAIHKNNPKSSLKINLQGLFIGNGLTDPENMIPMYGEVLYNLGFIDFNQRNEFENLQNLAVEAIRGEKWVDAAIYYAKLIIGYRFYPYPTLYTNLTGLETYYNYFAENSAKWLEFEEEFVRSAAFRGAVHVGDVARDDGRNTELFLMEDIAKSVIGWVEELVEHYKVVFYNGQLDLICAYPLTENFLRKMEWSGAEKYRNCSRRQWVVDGELAGYVRTVGNLSDVLVRNAGHMVPTDQPKWAYQLFNNFVHDRDW.	Down
**b39**	gi|91083421	NLU006778.1	0.597035289	0.570164323	26S proteasome non-ATPase regulatory subunit 7 *[Tribolium castaneum]*	TVGSLSQRVTNQLMGLKGLHLQLQDMRDYLNQSASLAVHATTNWHSSDFVTRRQSCTPSNWAAFLRVQVWWCMQQQTGTPQILSPDEKVARRQTGLLLNPDVISLYIGVLQTDTCWDYEDKVLFIFAEDIGVMLAGLYKVVEGKLPMNHQIVYQLQDIFNLLPDIGHGNFVDSLYVKTNDQMLVVYLAALIRSIIALHNLINNKLSNRDAEKKEINQKKEEKLKEDEVKKEVKPTKA.	Down
**b40**	gi|269995911	NLU019195.1	0.026791681	0.55462569	Yellow-c precursor *[Tribolium castaneum]*	DRLWVMDTGVANILGGGEQFTPAKILVYDLKTDELIRSFPIKSSDIKEDTFFPNIVVDVTKDKCDDAHAYIPDLGSNALIVYSWGKNDTWRIKHHYFNMDPLAGNYHVAGVNFQWVDGLFGIALSPVQEDGYRTAYFHALSSTREFTVSTRVLQNATEAESYYEYKPLGSRGPNSQATASMIDEKSGVLFYTQVNKNGIGCWNSVKHANEYSADTNGLVSSDNDTMVFPNDLKVDRDSNLWVLTDKLPLYIYRSLDNDDVNFRIFKAPVAEVIKGTVCDSK.	Down
**b41**	gi|328717285	NLU016607.1	0.824138105	0.809095919	39S ribosomal protein L10, mitochondrial-like *[Acyrthosiphon pisum]*	GYLASVKHLNDWCRLRDLETARSELVSVLNIGAQRIVADLSQQQRMLVGQLDQHVAINADKASAEGQQTGDHATQQSEAGDISVQTESQQNEAVKSAATENQQTSEAGKSAQSESQQSEAEKTAQASTGIVDGYLASVKHLNDWCRLRDLETARSELVSVLNIGAQRIVADLSQQQRMLVGQLDQHVAINADKASAEGQQTGDHATQQSEAGDISVQTESQQNEAVKSAATENQQTSEAGKSAQSESQQSEAEKTAQASSESTDPTNKTPGDS.	Down
**b42**	gi|306850727	NLU019790.1	0.779830098	0.544502676	Chemosensory protein *[Nilaparvata lugens]*	MFKNVLLVCLLVAVVSAKPKPAEKKQYTTKYDNIDLDEILNNQRLFDNYYKCLLGGKCTPDGQELREALPDALATACSKCTEKQRVGTEKVIKYLIEKKPTEYSELEKKYDPQGNYKRKYQAEAAKRGIKV.	Down
**b43**	gi|91079684	NLU024546.3	0.597035289	0.613762021	40S ribosomal protein S24 *[Tribolium castaneum]*	LNLQPIMDISKRTNVTISTIPYRYDKPLMNVNVNKLNSWLSGQISSSDDLDLLDLGHFEIGDYTRHGLHLNKSQGKKRIRKYIRNHLLGVGGHTSDTVVTLGKSHGGAQMARLDTEGTNNISSLGDDLNMTGDTDTDNTAAEELNNSFTRSNTHQHFLVNRSMSPQHGLYKKEKQTRKQRKERKNRMKKVRGTKKSKVGAAAGKKVNN.	Down
**b44**	gi|642926249	NLU021863.1	0.529663384	0.597035289	Mannose-1-phosphate guanyltransferase beta	MEALVAAGVKHVVLAVSYHAEEMEKELRIQAERLNVRLDFSHETEPLGTAGPLALAAEILNQTDEPFFVLNSDIICEFPFTELYEFHKNHKREGTIVVTKVEEPSKYGVVVYHADGCIKSFIEKPQEFVSNKINAGIYILNPSVLKRIEIRPTSIEKEVFPFMAEEGQLFAFELQGFWMDVGQPRDFLT	Down

					[Tribolium castaneum]	GMSMYLTSLRQKNASLLYNGPCAVGNVLVDPTAKIGVGCRIGPNVTIGPGVVIEDGVCIKRTPILRNAVVRSHAWLDQCIVGWRSVVGQWVR MENTTVLGEDVIVKDELYVNGGQVLPHKSIASSVPDPQIIM.	
**b45**	gi|641678830	NLU002926.1	0.717794299	0.235504895	Four and a half LIM domains protein 2 isoform X3 *[Acyrthosiphon pisum]*	MADVEVQQTTFTTEKKVRKVKKTSTSSKRRESVQDQDGGQQTTTVTITEMNKENHVSENGVQDQGYCDVWERNKIPRKSRGDLGDSSVIQ ECYELFGVVLMFLGGFH.	Down
**b46**	gi|193580101	NLU029038.1	0.613762021	0.586138189	40S ribosomal protein S9 *[Acyrthosiphon pisum]*	MVNKRIPSVFSKTYVTPRRPYEKARLEQELKIIGEYGLRNKREVWRVKYALAKIRKAARELLTLEEKDQKRLFEGNALLRRLVRIGVLDEGR MKLDYVLGLKIEDFLERRLQTQVYKLGLAKSIHHARVLIRQRHIRVRKQVVNIPSFVVRLDSQKHIDFSLKSPFGGGRPGRVKRKNSKKGTGG AAQDDEED.	Down
**b47**	gi|193638961	NLU010177.1	0.779830098	0.698232412	Neutral alpha-glucosidase AB *[Acyrthosiphon pisum]*	PEVTMPKDLMHYGGWEHRDIHNIYGMLFVMSTYQGRLNRGGVADKALAKRPFILSRSGFAGIQRYAALWTGDNAADWGHLAISLPMCLSL AISGVSFCGADVGGFFKNPDQELLIRWYQAGAFLPFFRAHAHIDTKRREPWLFGPEATSLIREAIRKRYMMLPFWYTQFYINNQTGLPVIRPL WAEFPADRNTFTIDNEFLIGDSILVRPVTEQGATEVKVYLPGQSEVWYDMDTYQPFQANGYINLPVGIRKTPVFVRAGSIIPLKLRVRRSSALM HNDPFTFVVVLKENGTAHGNLYIDDGSTFQFLYKKKSLFISLAFENLELSSKFIDKGHHYDTKSKVERVVIVNAPPSITRARATRLKAGQVTDL ETSYDSNNILTIRKPDLLMSEEWKITLH.	Down
**b48**	gi|6179938	NLU003099.1	0.855066717	0.824138105	Coatomer protein gamma subunit *[Drosophila elanogaster]*	YVQNPSPVAFDLKSIPPPSAVPDDHVTASNKIATEGSITSTVPKVSLASREESYAVKLQSVPEIAKLPGPLFRSSDPVQLTESETEYVVRCIKHSFP HHLVLQFECLNTLNDQLLENVWVQLEPSEGYQVIQSIPCPRLQYGESASTYIVLKFPDQLSATIGNFGATLKFIVKDCDVSTGLPDTEEGYDDE YMLEDVEVSLSDQIQKVTKANFGASWEEAASGFCEMEDMYSLASMTSLDEAVGNVTAFLGMQPADRSDKVPAGKSAHTLFLAGIFRGGHE VLVRAKLAMADAVTMQLTVRSTEQMVAELITSTVG.	Down
**b49**	gi|641659947	NLU009551.1	0.88715601	0.685488224	Partitioning defective protein 6 *[Acyrthosiphon pisum]*	QTVEVKTKFDAEFRRFSLPRSVPPKYDEFRSLIERLYRLSDVAFLISYTDPRDGDLLPINNDDNLARAIVSAKPLLRIIIQRKGESLEELNGYGTI KPRNLISTILGGTPGKSKGLCISNPHDFRQVSAIIDVDVVPEMCRRVRLLKHGSERPLGFYIRDGTSVRVTPDGLEKVPGIFISRLVPGGLAESTG LLAVNDEVLEVNGIEVAGKTLDQVTDMMVANSWNLIITVKPANQRTVAPPPRRGSFSRNSQLSSGSAQSSCPTTAGSDDDAYDRDEVVDLTA NLNDLPTAVTDKQGDNDDAILHL.	Down
**b50**	gi|157107343	NLU027817.1	0.862978518	0.642687678	Vesicle docking protein P115 *[Aedes aegypti]*	VSRHENYSTAFKQPQLRPRTPNDLLLDHEFCRLFKALEGMIIKAVNANASSGIANGGMDLSSSDNALVMQYKDLIREQDNKLRELEQENQVL RHENRTLTQQNEQLNSSMAQLRDQNMILRAQVNAGGSQVNQQEISLMVSELQTVKADCQRKDAVIQNLLEELHALKLSKMTITNEQNGQIN QPESNEETSGREAKLKWLLERETEERLNELISNSDELEQMKKDHDDLLELLADQDVKLSTYKDRLRKLGENVSPAKS.	Down
**b51**	gi|242247581	NLU020700.1	0.879022479	0.88715601	Transmembrane protein	MPPKKGKEGTKGQKQILEENASTLNFYRNMVFGANGIYLIFTFLLGDFFSLQTILLFMLSTATFVGSYQFLVFMSRPKLSETGQLLDGGLDLN	Down

					208 *[Acyrthosiphon pisum]*	MEGGLSEHFKDLIILTSGVQVLSLISNYFWFLWLLAPARGFWLLWKNILSPYFFQEAPAQEIDEKKQRKLERKMRRQ.	
**b52**	gi|193657389	NLU006634.1	0.01458814	0.870963573	Developmentally-regulated GTP-binding protein 2 *[Acyrthosiphon pisum]*	MGILEKISEIEKEIARTQKNKATEYHLGVLKAKLAKYRAQLLEPSKKSEKGEGFDVLKSGDARVALIGFPSVGKSTLLSTLTATQSEAASYEFTTLTCIPGVIEYKGASIQLLDLPGIIEGAAQGKGRGRQVIAVARTADLVLMMLDATKQTVQMNLLQKELESVGIRLNKQKPNIYFKVKKGGGIAFNSTCPLTKVDEKLVQMILHEYKIFNAEVLFREDCTADELIDVISANRVYLPCLYVYNKIDQISIEEVDRIARQPNSVVLSCNMKLNLDFLLDELWFYLALIRVYTKKPGQPPDFDDGLILRRGVTVEHVCHAIHRTLVSCFKYALVWGTSTKYSPQRVGLQHVMQDEDVIQVIKK.	Down
**b53**	gi|641666592	NLU011805.1	0.794328213	0.824138105	Protein PRRC1-like *[Acyrthosiphon pisum]*	AASNTAPAPLFHPTTFSPAIPADKASGEGVGVGKESGGAGAGAGEGGFGLMGWVRGAVDTSSSIVSKVAEKAKSSVDTMITTLDPQMKELLSPSVLFEVVVTSEEEIEISPIREAFHQIFGSQKVTVRGVRGTQEEECVAGAVSAVQPVGFEAAQQKAWKRIEALRAALQQPGPVVAVESFITELSPDKWYDVHAIVLSDSSNGIVVETFSQMTPIPSSIVTLAQDDTPADYQHKAAGVAVDIATIMARGRFSVHIATIMASNLHVHESEWHHALTGVSRRDMILLAAKSLVNIYKNSLQ.	Down
**b54**	gi|193580274	NLU023863.1	0.103752799	0.369828194	Signal recognition particle 54 kDa protein *[Acyrthosiphon pisum]*	FEIIIVDTSGRHKQEESLFEEMLQVSNAIRPDNIIFVMDATIGQACEVQARAFKEKVDVGSVIVTKLDGHAKGGGALSAVAATNSPIIFIGTGEHIDDLESFKTKPFISKLLGMGDIEGLIDKVNELKLDDNEELIEKIKHGQFTLRDMYEQFQNIMKMGPFSQIMGMIPGFSQDFLPKGSEQESMARLKKLMTIMDSMNDGELDHRDGAKLFSKQNGRVVRVAQGSGVTEKEVKDLISQYTKFAGVVKKMGGIKGLFKGGDMAKNVNQAQMAKLNQQMAKMMDPRVLHQMGGMSGLQNMMRQLQQGAAGAAGGLSNLMGGFGGKS.	Down
**b55**	gi|21356979	NLU018325.1	0.824138105	0.704693079	CG1910, isoform A *[Drosophila elanogaster]*	MSESSSVEVKNESSNDVDSKAPESTPARGRKNRLSMLSRAALEGEAILKGMGHKEDGTTEGRRTTRSSTRGTPTPVATPPPAKRERKTASTGSSGRGGRRGRPRKTEPVDNNDDGKDEEKEDKKEEIGENGEKQNNSEDAESTEADEKMETDSAANETSKKEDSKSTSLDNGPTTAAKSEKKEDAAEPPAKPVEPPKEEANAKKEEKKEEKEESPKKEPAAATAPQTAAEQDNEKKEEKAKEANEKDEKKSEQPDVKSSPSSKESAAAAPPAQPASIEQPAQ.	Down
**b56**	gi|641668185	NLU014710.1	0.787045777	0.855066717	Uncharacterized protein LOC100572041 isoform X3 *[Acyrthosiphon pisum]*	LSHKLKTLRRCAVEVGLDGIEYKGLSPDAFNYLIQLAKLKELLKITFEDLKLVQLTKVSDNTDINKEIGKLFKPNKSQTHDTGTRQKQSDVFAMRTFPKRADLPEPDRDVILRIGETQKLMVPYVPPFKGVARDGRVEVVAFNANKKAITKYTLQLRKQMTDYIASQPTNRYVPRGGELCVCLQDGKENEKIQKISSTGCIPANTTFEELKMVFDEIFEAKIKRFNDEHYTFYSPEIIARLTNKLK.	Down
**b57**	gi|345495054	NLU025070.1	0.824138105	0.879022479	Polyadenylate-binding protein-interacting protein 1 *[Nasonia vitripennis]*	DDIAVYQPTNHRTFFIDENVVHNLFQNSAYRDSSEDGININTTDNEIGRTIDYIVRQLLVNPGSLDDLMKVLKDDIKRWGFRDVTIAFIMTCIIDATISLDNFAYLGAKICCHLDKIVSKLRPNISIRTLVLTACEDKFSEFENWLLAEGTRNTAYNFIFFLAELYDQLLVQNARIKALWEALCGTYIILMKDPNNSFSIRCLCKVLKLTGRNLYQDNRLMLQGIIAQLEEISRSNMFDSQTAAMLSSVIGLANSHWGLDESQLTTNHIDSAHRSEGRGAEMYGPDGRVLTTDEKKFLADHCLEVAEDEEEYGLQDSYVDSDDGMDEEMQEAFEQFLLSVPEAR.	Down

**b58**	gi|641669441	NLU020524.2	0.879022479	0.895364821	Ubiquitin carboxyl-terminal hydrolase 4-like isoform X2 *[Acyrthosiphon pisum]*	QMSANPKCTICFEEKEANMKRHLGCDCVLCDACVTTSCEHYGKDGMVCPVCRLKLDPDIELVAVEKSLPSWPTVRMLHVPVVYRLDTLGDGNNNKKTVSLFGHPGLLRLPNNISCQSLYSVIASINPYEGDFKILLVDGQGRHCSRCMFNSHCRGCVVEGEGEGEDGGPVHLRTGDTLAVTFTEPVSELHVSKHESVAALRSQQPLRIYDCIQAFSQSEVLDKQNPWFCPKCQKNQCATKTLSIWRYPDYLIVYLKRFVFHDGVSTKLEDKVIFPIHGLNLSPYTSLYDLYACVCHIGGVSAGHYTSYTQHPQTGEWHYYNDDYVTKQMPQEEDYSNAYILFYKKRGYVGTVFSYALAILNLVFRPVSTVVMGKNCANRMGLSGGFYPATFGTIFVDCLGFDLTQRQDGMSPTAVGGGQTVPRI.	Down
**b59**	gi|332021445	NLU020570.1	0.046558611	0.212813899	Putative protein phosphatase *[Acromyrmex echinatior]*	SDDDDDEDSEDLDFENAQASDDDDDDEGDSVAVEMDDEDGEDDEDEDDDDEEEDEDDDECDGRFSVLPEEPGMDSGCTAVVALLAGNSLYVANAGDSRCIVCRNGEAIEMSIDHKPEDAPEMERILAAGGTVKEGRVNKGLNLSRAIGDFTYKKNSALPPTEQMIIALPDVKKLEVDTAQDEFMVLACDGIWNSMTSQEVVDFVKPKLHEGVPVSKICEQMFDHCLAPNTLGDGTGCDNMTCIIVKFKSNDSPLEVPPVVKRRCASPDDQECQDRPKRQKTDESLTGSD.	Down
**b60**	gi|746861074	NLU026070.1	0.809095919	0.751622915	Dehydrogenase/reductase SDR family protein 7-like isoform X1 *[Acromyrmex echinatior]*	MSEMDGPGWTFLWWMLTSVFIPISIPYLVYKIFYSKTTKYKLQGKVVLITGASSGLGEALAHSFYKAGCKVILTARREKELARVKNDLLSLHPTVPTYPPVALLLDISKLEDIPKYVSEVLAIHKNVDILVNNAGISYRGDVMSTKLEVDEQVMAVNYFGTVALTKALLPQMVEGNGGHIVAVSSVQGRIAVPHRSAYAASKHALQAFFDSLRAELAGSNVKVTVVSPGYIKTNLSLNAVTGSGEVYGEMDETTASGYSPEMVANKIVVSVAKQKNEVVIAPLSARIGIGLRTVLPSLYFRIMERRAAKAAKKK.	Down
**b61**	gi|328713406	NLU011024.1	0.691830993	0.758577585	Ankyrin repeat and FYVE domain-containing protein 1 *[Acyrthosiphon pisum]*	MECFKDLYSLTPSSSPQSGNRRIVKISYLRWGFASVMVHRLSHTVTVGNEINKREQHLSLLKEEYVKLQSYCSDLERKYALASASIGDLNENSFVSSLLKTVTSLHKNTLFSDMKVKLEDHVVPAHKIVFASRNSTWGISKSIEQIDILDWSHLGKDVGSAVLKWVYTDQIDFSKGDDFTLSLMKTANDFKLEEVVSKSEKALMASVNVKNCVRFYSTADEIGAETLKEHCSSLISANWDRLRDMGSASVTGSSIIECRKKHPITDDSSRKRPLLCGGRAGAHPGDKYNNWEPRVLFLACRLVRGYVRAPDPLTAGTAQCPDRGARSIGVVPLGGSYRLNSSVILGYRLGSAVM.	Down
**b62**	gi|270047496	NLU004616.1	0.870963573	0.765596628	U1 small nuclear ribonucleoprotein A *[Apis mellifera]*	MDIRPNHTIYINNLNEKIKKEELKKSLYAIFSQFGQILDIVAMKTLKMRGQAFVIFKEIASATNALRSMHGFPFYDKPMRIQYSKVDSDVIAKMKGTFAERPKKASRKAAAAAAAAGGAAAAAALEELAAESKKSKRKAAKEQARIYQQQQQQSMQQMASAAPPMMAVGGPTTVPEQPPNQILFLTNLPDETNEMMLSMLFNQFPGFKEVRLVPNRHDIAFVEFETEMQSGAAKIALNGFKITPSHAMKITFAKK.	Down
**b63**	gi|478346216	NLU002797.1	0.816582382	0.879022479	Tropomodulin *[Nilaparvata lugens]*	PNATDGDKTIQQVGDDDHNLVDLNWNNIKNISDEKFEQLFSALGDNTHLETLSLVNVNLNDRTAIKLADAIEKNNTLRVLNVETNFISPVGIVHLVKALLNQKVIEEFRASNQRSQVLGNKIEMEITKLIEQNPTILRLGLHLEYNDARHRIATHLQRNIDRNMRALRKATALETEASFTLGRQQDGSLHIIAQA.	Down
**b64**	gi|401879814	NLU021861.1	0.870963573	0.879022479	GTP-binding protein Rheb	MRLRSQDYDLKLVDTAGQDEYSIFPAQYSMDIHGYVLVYSITSSKSFEVVQIIYDKLLDMTGKIHVPIVLVGNKTDLHLERMISAEEGKKLADTWKAAFLETSAKQNEAVADIFHTMLLEIEKANGNMQEKASCVIS.	Down

					[Nilaparvata lugens]		
**b65**	gi|215254082	NLU021840.1	0.724435985	0.870963573	Putative chemosensory protein CSP6 *[Nilaparvata lugens]*	MTGNNVNIIYSTVPQITQYRVQSTSYKMLWAARFIVLPLLFCVLQVWSAPADEKYTDIDFDSILANRRVLSSYVKCLTDKGPCTPQGKELKKIVPEVIQTSCTKCSPQQKKVVRNVITTMQSKYKDQWDLVVNKYDPKKQRSGELKAFLSGTD.	Down
**b66**	gi|187113154	NLU025556.1	0.01213389	0.801678121	Cell division cycle 42 *[Acyrthosiphon pisum]*	MQTIKCVVVGDGAVGKTCLLISYTTNKFPSEYVPTVFDNYAVTVMIGGEPYTLGLFDTAGQEDYDRLRPLSYPQTDVFLVCFSVVSPSSFENVKEKWVPEITHHCQKTPFLLVGTQIDLRDDAGTVEKLAKNKQKPISFEQGEKLAKELKAVKYVECSALTQKGLKNVFDEAILAALEPPEPAKRKRCVLL.	Down
**b67**	gi|193620426	NLU008089.1	0.672976673	0.01213389	Protein translation factor SUI1 homolog *[Acyrthosiphon pisum]*	MNQAQDDVMADNNQYTKASKDGLAVGKIMVVACYDFHIPLLQPQHISSPCQVKKKKKQSMGDVVMEDRVEATKMDLKATPEVALPSNPRVCPIRAVSKGKKEKPKRPEIGETPGTNVGHEAESRVTGHRAQNFLGEVRLLTLQFRRGKKKIPKIPMMSEKTGTNVGKEAESRVAGRPSGNLAAPSSAEGPTKKARSGTHVDPFADAIKGSEDDVQDGLVHIRIQQRNGRKTLTTVQGLSSEYDLKKIVRACKKEFACNGTVIEHPEYGEVLQLQGDQRENICQWLTKTGLAKPDQLKVHGF.	Down
**b68**	gi|157134829	NLU017575.2	0.809095919	0.809095919	AMP dependent coa ligase *[Aedes aegypti]*	MYIVPPIMLFMTAHPAVTKKQLEHMRFLASGAAPIGASDVTRTLHKMPESCIFMQAYGLTETSPVCLLPRNNNRNLSTVGSPTSITKAKIIDTSTGKILGPNEHGELCIYGPQVMQGYLNNQKATDETIINGWLHTGDIGYYDDEGLFYIVDRLKELIKVKGFQVAPAELEELLRTHPKVNDVAVIGVPDARSGEVPLAYVIRKDDVTEDELKGYISDRVAPFKQLAGIVFTDSIPKSPSGKILRRFLKDAYLKEHKK.	Down
**b69**	gi|642938505	NLU008801.1	0.809095919	0.816582382	Von Willebrand factor A domain-containing protein 8-like *[Tribolium castaneum]*	MLARGAFEHAKIPTGFRFAHMPQRTVSTRANASPYTYAHPRQTYRVPASNGHSYHIVIQKYSLSIYVTRTAEGHLNLDQETLHHLRWMLQKDLLGQDMFLIGRPGPLKRRLALQYLQLTRREFEFVALSRDTTESDLKQRREIMAATAKYIDQGAVRAAVNGRVLILEGIEKAERNVLPVLNNLLENREMHLEDGRFLVPASRYDKLLQNNRLWSGHTYLHRARAAARPSDKYDCEASEKTNAFKRVGTHRYYRTASALSPCLMPDRARAVTV.	Down
**b70**	gi|642938243	NLU009216.1	0.685488224	0.779830098	Serine/threonine-protein kinase Doa isoform X1 *[Tribolium castaneum]*	KYKVEARHAHAQAKTSLYDFHKELGGKFVNFGGYELPVQYAAEGIAASHLHTRASCSIFDVSHMLQTEVRGSDRIAYFESLCTADVQGLPENGASLSLFIDDSTGGILDDLIVTKAASHLFIVSNAGRRYHDSQLMIRTQDEFRKQGKDVTVRFLSPEKQSLLAIQGPRTAEILQPFTDIDLEKLYFMKSEIGTVCGIEDCRVSRCGYTGEDGVEISVKSERAPFIVSKLLKKYPEVVKMAGLGARDTLRIEAGLCLYGNDIDETVTPAQAGLGFTVAKRRRQMKDFNGAPIILSELEWGPYKKRVGLKSLGK.	Down
**b71**	gi|641673885	NLU022606.1	0.01137627	0.648634374	Uncharacterized protein LOC103310331 *[Acyrthosiphon pisum]*	AEYIVQEASRLTEIFEIAHPYMILEVASMALHEENGELAVELLRYLVDSVSVDHKLCTKLLDFVGEHIFNLTKYMCNTSKPFEIASLLKTVSSMCLESASVVSLGYEGTNFQLGSDELNAKGLVSISQDGGKHDFKVGEMHTVRVIHYDLMENVYICSMKPRRLGWAAAGDGRLGVVSVNHPRSSRGLKQGLEYLVEVIPSFRKDRSRAGRDRSRNDLVSSVSMVSGTGEAFPIVTGCLLAIPDGISKVEEPRNWASRLVTGSLTSGTGYFWFSGSVSRGRARPS	Down

**b72**	gi|157138048	NLU010460.1	0.724435985	0.879022479	4-Hydroxybutyrate CoA-transferase, putative *[Aedes aegypti]*	MKTATSPRIRESQFSDSSCLKSRTWLNPELGNRPRFGGQVDFIRGASEALDGKGKPIIAMTSTNPKTGDSKIVPFIKEGAGVVTSRAHVHYVVT EQGIASLFGKSLRQRAYHLIEIAHPTHREALEKAAFKRLKCMPAP.	Down
**b73**	gi|641677178	NLU026673.1	0.731139123	0.066680677	DNA-directed RNA polymerase II subunit RPB1-like *[Acyrthosiphon pisum]*	VLMDAASHAEVDPMRGVSENIIMGQLPRMGTGCFDLLLDAEKCKEGFEISASMGVGMMDGSGMYFGSAATPSMSPSRTPWQHAGTPAYGV TAWSPGEGSGMTPGAPGFSPSGSSDASGLSPGYSPGWSPTPGSPGSPGPSSPFIPPTVGGMSPSYSPTSSTYAPASPMGAGTAGSLVAARLAEDA DVSVLVLEAGGAPSPLF	Down
**b74**	gi|641672130	NLU004779.1	0.831763685	0.032809529	Sorting nexin-6 isoform X3 *[Acyrthosiphon pisum]*	LYRRLRCLSNYEAANRNLEKARAKNKDVLAAETAQQEACEKFEKMSDQGKEELMDFKQRRVQQFRKNLIELAELEIKHAKSLGISYYCSGF LEEVLNLVSEVSMQLALHGQSSDVESISLENPELLNENELVHRRDGSKRALVYLIQHDGAPAHFHLEARDFLDNCFQHRWIGRAGPIAWTPCS PDFNTDGFLPVGFQRFIKDKIYVPSLPDNLDDLRNRITIISVDKLRLICWYECGKKRNRLLVGCMLHYQWLSHRTKTSPTPQT.	Down
**b75**	gi|641679116	NLU008489.1	0.724435985	0.679203629	Uncharacterized protein LOC100160882 *[Acyrthosiphon pisum]*	SLKMPPPSYLKDGAQRNENILRMNEIQERFTQPRVRLVQSPWEAALHTGSVEAAFQEVPSRGTWREALSRSPVIFDALAGGGPPLAPPRTISPA PAYQSPLPPANVDVYKPKVPQGWAAPSAPRKLPQHQPIGNLKLEKLFAGRPKTPITLVYNAYDDTLSTSAANEPQPPPKPPSPELPEPSEYLKF MSEFTKKNSSNNTDSSNLQTSTTQNVQFSGNNSSQQFSNNNAQFDNSGSNFQQTPQFSSIGNTSSLGSSETVANVSKTIYKQEVVESEETTESM CFKNIEKFEKNSICLGAQEGMIEKNAE.	Down

Supplementary Table 2.The information of the genes/proteins up- or down-regulated at both mRNA and protein level

No.	Accession No.	Unigenes No.	Protein name	GSi / GFPi Regulated
**a1**	gi|157133341	NLU001572.2	Citrate synthase*[Aedesaegypti]*	Up
**a2**	gi|193659676	NLU022227.1	Phenoloxidase subunit A3-like*[Acyrthosiphonpisum]*	Up
**a3**	gi|339765122	NLU011146.1	Cytochrome P450 CYP6ER1*[Nilaparvatalugens]*	Up
**a4**	gi|156542034	NLU025371.1	Peroxiredoxin 1*[Nasoniavitripennis]*	Up
**a5**	gi|642930478	NLU022689.1	Aodium/potassium-transporting ATPase subunit alpha isoform X3 [Triboliumcastaneum]	Up
**b1**	gi|170056920	NLU001990.1	Disulfide isomerase*[Culexquinquefasciatus]*	Down
**b2**	gi|91087369	NLU017666.1	Translation elongation factor 2*[Triboliumcastaneum]*	Down
**b3**	gi|540361100	NLU026472.1	Arginine kinase *[Nephotettixcincticeps]*	Down
**b4**	gi|723001888	NLU019204.1	Vitellogenin*[Nilaparvatalugens]*	Down
**b5**	gi|641676959	NLU016669.1	Aldehyde dehydrogenase, *[Acyrthosiphonpisum]*	Down
**b6**	gi|91083095	NLU014434.1	40S ribosomal protein S4 *[Triboliumcastaneum]*	Down
**b7**	gi|478896864	NLU012724.1	Glutamine synthetase 2 *[Nilaparvatalugens]*	Down
